# Pharmaceutical 3D Printing Beyond Printability: Polymer Functions, Structural Design, and Predictable Drug Release

**DOI:** 10.3390/pharmaceutics18101330

**Published:** 2026-10-20

**Authors:** Zhiting Liu, Yi Huang, Yanpai Liu, Rui Huang, Peixuan Xie, Xinyao Ma, Peihong Chen, Xiaofang Li

**Affiliations:** 1 School of Traditional Chinese Medicine, Guangdong Pharmaceutical University, Guangzhou 510006, China; 2 State Key Laboratory of Quality Research in Chinese Medicine, Institute of Chinese Medical Sciences, University of Macau, Taipa, Macau 999078, China; 3 Guangdong Provincial Key Laboratory of Pharmaceutical Preparations Research and Evaluation & Guangdong Provincial Engineering Center of Topical Precise Drug Delivery System, School of Pharmacy, Guangdong Pharmaceutical University, Guangzhou 510006, China; 4 Key Specialty of Clinical Pharmacy, The First Affiliated Hospital of Guangdong Pharmaceutical University, Guangzhou 510080, China; 5 Guangdong Pharmaceutical University, Guangzhou 510006, China; 6 School of Biomedical and Pharmaceutical Sciences, Guangdong University of Technology, Guangzhou 510006, China

**Keywords:** 3D printing, additive manufacturing, polymer, oral solid dosage forms, 3D-printed medicine, quality by design

## Abstract

Oral solid dosage forms, due to their mature manufacturing processes and stability, have become the cornerstone of clinical drug therapy. However, fixed dosages, uniform components, and large-scale production may not meet increasingly complex personalized requirements. The emergence of 3D-printed drugs provides a potential solution to this challenge. Drug 3D printing has progressed beyond feasibility verification and is moving toward functional dosage form design. Current research primarily focuses on printability; however, this review integrates printing technology, polymer functionality, structural design, release behavior, and translational requirements, summarizing mainstream printing technologies, including Fused Deposition Modeling (FDM), Direct Ink Writing/Semi-Solid Extrusion (DIW/SSE), Powder Bed Printing (SLS/BJ), and Photopolymerization (SLA/DLP). Polymers are classified according to drug release control functions, illustrating how modulation of porosity, filling density, shell thickness, material partitioning, and spatial drug distribution enables 3D-printed dosage forms with diverse therapeutic functions. These functions include immediate and sustained release, gastric retention, intestinal targeting, and sequential release of multiple components. This review provides guidance for technical selection, structural design, and formulation development of 3D-printed drugs, while highlighting major translational barriers, including limited printable excipients, empirical formulation development, insufficient structure-based release prediction, quality control challenges, and weak clinical evidence. Future progress requires close integration of material, process, structure, and performance, enabling 3D-printed drugs to evolve from individual prototypes into scalable, quality-controllable, and clinically translatable drug delivery systems.

## 1. Introduction

Oral solid dosage forms remain central to clinical pharmacotherapy due to their manufacturing maturity, stability, and patient acceptability [1]. However, formulations with fixed doses, homogeneous compositions, and large-scale production may be inadequate for specific clinical scenarios, particularly in pediatric and geriatric patients, individuals with dysphagia, patients requiring long-term therapy, and those needing flexible dose adjustment, multi-drug regimens, or tailored release profiles [2,3,4]. Therefore, a major challenge in modern formulation science is how to translate the flexibility of material, process, and structural design into predictable release performance and quality-assured manufacturing [5].

Pharmaceutical 3D printing provides a formulation-manufacturing approach in which digital designs can be directly translated into the geometry and internal architecture of dosage forms [6]. By regulating external morphology, internal density, pore structure, shell thickness, material partitioning, and spatial drug distribution, 3D printing enables the design of dosage forms based on wetting pathways, diffusion distances, exposed surface areas, and release kinetics [7]. In this context, structure serves as an active formulation variable rather than a passive container, as the same material system can exhibit distinct disintegration, dissolution, and release behaviors when fabricated with different geometries, infill patterns, pore networks, or multi-zone architectures [3,8,9,10,11,12].

Over the past decade, pharmaceutical 3D printing has evolved from proof-of-concept demonstrations toward functional formulation development and early-stage translational evaluation [13]. The approval of Spritam^®^, a porous 3D-printed levetiracetam formulation designed for rapid dispersion, demonstrated the commercial feasibility of printed pharmaceuticals [14]. However, Spritam^®^ mainly highlights the feasibility of producing porous, rapidly dispersible printlets and does not, by itself, address the broader challenge of establishing predictable relationships between complex printed structures and drug-release performance. Recently, printed drug products and manufacturing platforms have been increasingly investigated in preclinical, translational, and regulatory-oriented studies, reflecting growing interest in their clinical and industrial applicability [13].

Despite these advances, the central question in pharmaceutical 3D printing is shifting from whether a formulation can be printed to whether its material composition, process parameters, and structural features can enable predictable, reproducible, and clinically relevant drug-release performance [5]. Each printing technology imposes a distinct material processing window, including requirements for thermoplasticity, rheological properties, powder flowability, energy absorption, or photoreactivity; however, most pharmaceutical excipients were originally developed for conventional processes such as compression, coating, or capsule filling rather than layer-by-layer manufacturing [15,16,17]. Moreover, drug release from printed formulations is governed not only by polymer hydrophilicity, hydration, dissolution, and degradation, but also by structural variables such as infill density, porosity, shell thickness, surface area-to-volume ratio, and multi-material spatial distribution. As structural complexity increases, formulation development often remains empirical, and transferable design principles linking material properties, process parameters, structural features, and release kinetics remain insufficiently established [17,18,19,20].

Building on previous reviews of printing technologies, materials, and personalized medicines, this review links polymer functions and processing constraints to structural variables and experimentally observed drug-release behavior. It examines how these relationships guide formulation design and what evidence is needed for prediction and translation. The primary focus is oral and oral-related dosage forms, with implantable and localized systems considered as extensions. Study selection and evidence interpretation are described below. Literature Search Strategy: To provide a comprehensive narrative overview of pharmaceutical 3D printing, the relevant literature was retrieved from PubMed, Web of Science, and Google Scholar. The search period primarily covered publications from 1990 to April 2026, supplemented by representative high-quality reviews and key original studies. The search terms included, but were not limited to, “3D printing”, “additive manufacturing”, “pharmaceuticals”, “drug delivery”, “polymers”, “fused deposition modeling”, “hot-melt extrusion”, “semi-solid extrusion”, “selective laser sintering”, “binder jetting”, “stereolithography”, “digital light processing”, “printability”, “drug release”, and “personalized medicine”. The selected literature primarily focused on pharmaceutical 3D-printing processes, polymer material selection, formulation structure design, drug-release control mechanisms, and advances in clinical translation. Representative original studies were selected when they provided sufficient information on the printing method, formulation or polymer matrix, model drug, dosage-form design, and experimentally characterized release behavior. Studies were further selected to represent different printing platforms, material systems, structural strategies, and release functions, while avoiding excessive duplication of highly similar formulations. Representative studies were selected when they clearly demonstrated a relationship between defined formulation or structural features and the intended drug-release function, covering immediate/rapid release, sustained/controlled release, gastrointestinal site-specific delivery, and multicomponent or sequential release.

## 2. Pharmaceutical 3D Printing Technologies: Process Windows and Formulation Compatibility

**Figure 1:**
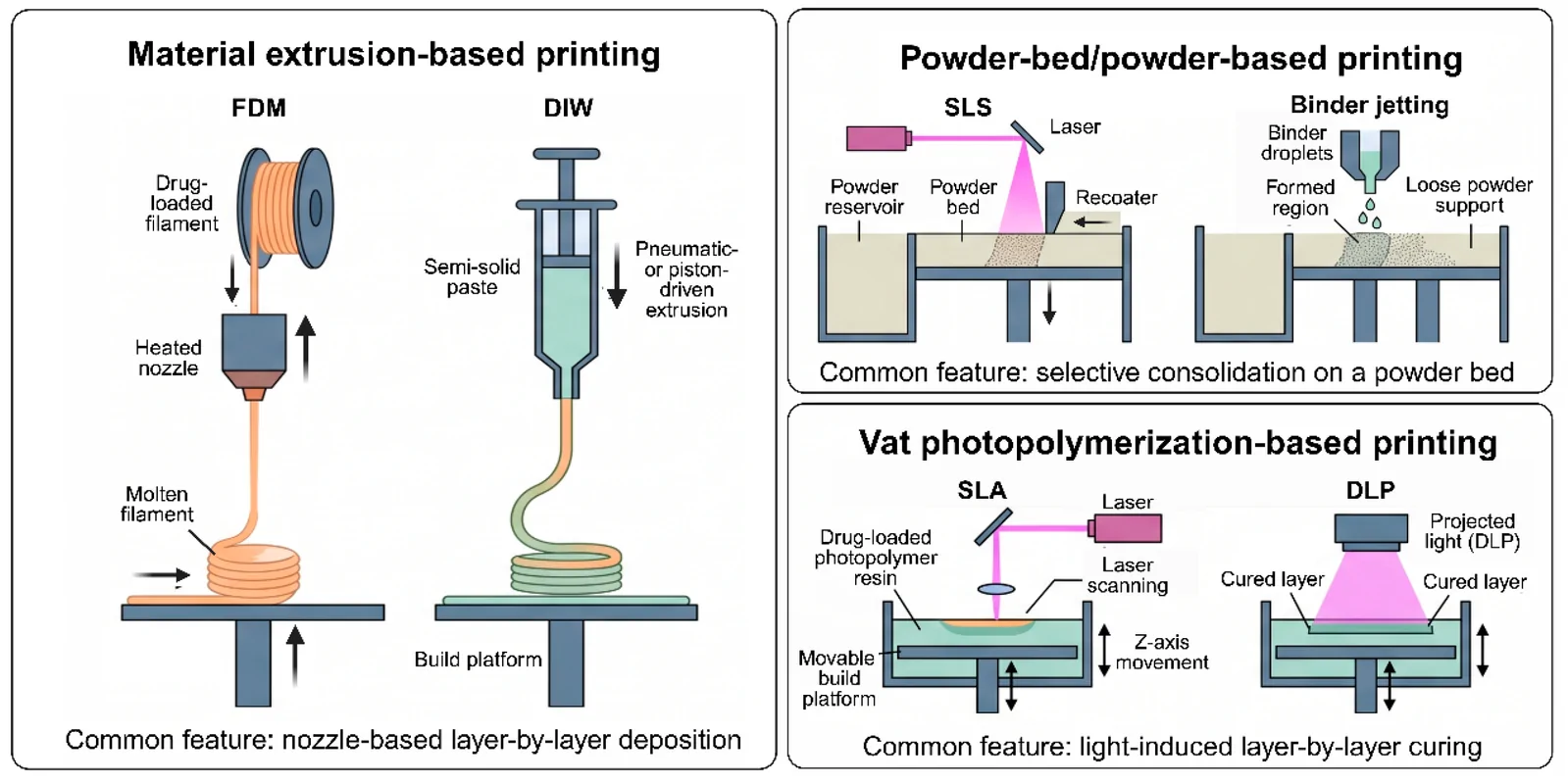
Schematic illustration of three mainstream 3D printing categories for pharmaceutical applications. Material extrusion-based printing includes representative techniques such as fused deposition modeling (FDM) and direct ink writing (DIW), both of which fabricate dosage forms through nozzle-based layer-by-layer deposition. Powder-based printing includes selective laser sintering (SLS) and binder jetting, which rely on selective consolidation of powder beds to construct dosage forms. Vat photopolymerization-based printing includes stereolithography (SLA) and digital light processing (DLP), which generate structures through light-induced layer-by-layer curing of photopolymer resins. These representative pharmaceutical printing technologies are classified into three major additive manufacturing categories.

Pharmaceutical 3D printing should be considered a family of formulation-manufacturing technologies rather than a single preparation method. Each printing process defines a specific material window, including feedstock form, energy input, solidification mechanism, post-processing requirements, and potential quality risks. In pharmaceutical formulations, technology selection is not simply an equipment-related decision but represents the starting point of formulation design. This choice determines whether the drug maintains stability, the material remains processable, the printed structure achieves sufficient integrity, and the intended release profile can be reproducibly obtained. The three major categories of pharmaceutical 3D printing technologies and their representative manufacturing processes are schematically illustrated in Figure 1.

Based on current research and translational progress, pharmaceutical 3D printing can be broadly categorized into material extrusion, powder-bed printing, and photopolymerization-based printing technologies [5]. Material extrusion technologies include FDM and DIW/SSE [3,21]. Powder-bed printing includes binder jetting (BJ) and selective laser sintering (SLS), which are particularly suitable for fabricating porous structures and support-free architectures [22,23]. Photopolymerization-based printing, represented by stereolithography (SLA) and digital light processing (DLP), offers advantages in producing high-resolution microstructures, microneedles, and localized delivery devices [24,25]. The applicability of each technology depends on drug properties, excipient characteristics, target dosage form, and release requirements [15].

### 2.1. Material Extrusion Printing: FDM and DIW/SSE

Material extrusion is the most extensively studied category of pharmaceutical 3D printing and produces printlets through nozzle-based deposition of softened or flowable drug-containing materials [26,27]. Although FDM and DIW/SSE both rely on path-controlled layer-by-layer deposition, they differ fundamentally in terms of feedstock characteristics, processing stress, and formulation constraints [28]. FDM generally requires drug-loaded thermoplastic filaments prepared by hot-melt extrusion (HME). Therefore, polymer glass transition or melting behavior, melt viscosity, filament mechanical strength, drug thermal stability, and interlayer adhesion collectively determine printability and the quality of final products [16,29]. FDM is particularly suitable for fabricating mechanically robust printlets, controlled-release formulations, gastroretentive systems, polypills, and complex architectures containing shells, channels, or internal compartments [30,31]. However, its application remains limited by thermal processing requirements, potential drug solid-state alterations, filament brittleness, feeding instability, and restricted drug-loading capacity. Fused filament fabrication (FFF) is broadly equivalent to FDM and is also used to describe filament-based material extrusion in pharmaceutical studies. Other related approaches reported in pharmaceutical manufacturing include Arburg Plastic Freeforming–Droplet Deposition Modeling (APF-DDM), a thermal droplet-based freeforming process, and direct extrusion additive manufacturing (DEAM), a filament-free material extrusion approach. Injection volume filling (IVF), although not itself an extrusion-printing process, has also been combined with printed cavities or scaffolds to introduce drug-containing formulations after fabrication.

DIW/SSE uses gels, pastes, slurries, or semi-solid inks that are extruded through a nozzle under pneumatic, piston-driven, or screw-driven pressure. Compared with FDM, DIW/SSE operates under milder processing conditions and provides greater formulation flexibility, making it particularly suitable for heat-sensitive drugs, high drug-loading systems, orodispersible formulations, and small-batch personalized manufacturing [28,32]. However, DIW/SSE shifts the primary formulation challenge from thermoplastic processability to precise rheological regulation. Parameters including ink viscosity, yield stress, shear-thinning behavior, structural recovery, drying shrinkage, and solvent removal critically affect dimensional accuracy, interlayer integrity, dose uniformity, and release reproducibility. Thus, FDM performance is primarily determined by thermal processing parameters and filament properties, whereas DIW/SSE relies more heavily on ink rheology, shape retention, and post-processing stability [33,34].

### 2.2. Powder-Bed Printing: BJ and SLS

Powder-bed printing fabricates dosage forms by selectively consolidating powder layers through binder-mediated bonding or laser-assisted sintering [35,36]. Unlike extrusion-based printing, powder-bed processes rely on powder spreading and localized consolidation, while the surrounding unsolidified powder can serve as temporary support during fabrication [36,37]. These characteristics make powder-bed printing suitable for producing porous, rapidly wetting, or support-free structures, but also introduce quality challenges associated with powder flowability, layer uniformity, powder removal, powder recovery, dust control, and mechanical robustness [38].

BJ forms printlets by depositing binder droplets onto powder beds, producing highly porous and low-density structures that facilitate rapid medium penetration and disintegration. The commercial example of Spritam^®^ highlights the translational potential of BJ in the development of rapidly dispersing oral formulations [35,39]. However, the dependence of structural integrity on localized binder-particle bonding may result in insufficient mechanical strength, increased friability, uneven binder distribution, and difficulties in maintaining dose uniformity. Therefore, effective management of powder handling, de-dusting, powder recycling, and cross-contamination control remains essential for the scale-up and industrial implementation of the technology [38,39]. SLS consolidates polymer powder beds through localized laser-induced softening, melting, or sintering processes. Compared with BJ, SLS generally achieves higher structural integrity without the use of liquid binders; however, the applied laser energy may also affect powder-bed characteristics, including porosity, drug dispersion, crystallinity, and subsequent release behavior [37,40]. Owing to these features, SLS is suitable for fabricating porous printlets, orodispersible systems, sustained-release structures, and formulations in which controlled powder sintering is exploited to regulate dissolution. Nevertheless, thermal exposure during processing, variations in powder spreading, potential thermal degradation, and the requirement for light-absorbing agents may compromise drug stability, excipient safety, content uniformity, and release reproducibility [40]. Overall, BJ is more advantageous for highly porous and rapidly disintegrating formulations, whereas SLS is more appropriate for solvent-free powder-based structures that require enhanced mechanical integrity or sintering-mediated control of drug release. For both technologies, major barriers to clinical and industrial translation include powder processability, powder recovery, product robustness, batch-to-batch consistency, and control of the manufacturing environment [35,38].

### 2.3. Photopolymerization Printing

Photopolymerization-based printing, including SLA and DLP, fabricates three-dimensional structures through light-induced polymerization or crosslinking of liquid resins, prepolymers, or hydrogel precursors containing photoreactive groups [41,42]. Compared with material extrusion and powder-bed printing, the primary advantages of photopolymerization-based technologies are their high resolution, excellent surface quality, and strong microstructural fabrication capability. These characteristics make SLA/DLP particularly suitable for applications such as microneedles, ocular inserts, implantable devices, drug-eluting structures, and hydrogel-based localized delivery systems [43,44]. However, their application in high-dose oral solid dosage forms remains limited, mainly because most photopolymerization systems require photoreactive monomers, oligomers, crosslinkers, and photoinitiators. The presence of these components raises concerns regarding residual toxicity, incomplete curing, potential drug instability, and long-term biocompatibility [45].

For drug-containing systems, photopolymerization enables the regulation of drug release through adjustments in crosslinking density, network pore size, hydrogel composition, and device geometry. This feature makes photopolymerization-based printing particularly suitable for localized or microstructured delivery systems, in which drug release is primarily controlled by diffusion through a crosslinked network. Nevertheless, factors including drug loading, light penetration, resin viscosity, oxygen inhibition, curing shrinkage, post-curing treatment, and residue removal can significantly affect product quality and release reproducibility [42,46]. Emerging technologies such as CLIP and TPP may further enhance printing speed or microscale resolution; however, their pharmaceutical applications remain largely limited to research-stage microstructures and localized delivery devices [41,47,48,49]. Volumetric additive manufacturing (VAM), particularly tomographic volumetric printing, represents another emerging photopolymerization approach with potential relevance to pharmaceutical manufacturing. Unlike SLA and DLP, which construct objects sequentially layer by layer, VAM generates three-dimensional structures within a photosensitive resin by delivering multiple angular light projections that collectively exceed the polymerization threshold only within the target volume. This layerless fabrication strategy can substantially reduce manufacturing time; drug-loaded printlets have been produced within seconds, and simultaneous fabrication of multiple tablets has also been demonstrated. Such rapid processing may be advantageous for on-demand or point-of-care production of personalized medicines, while the absence of sequential layering may reduce layer-associated defects and broaden the processing window for certain viscous formulations [50,51]. However, pharmaceutical applications remain at an early stage and are constrained by requirements for optically suitable and photoreactive formulations, limited availability of pharmaceutically acceptable resin systems, light scattering or absorption by drugs and excipients, residual monomers and photoinitiators, and the need to establish reproducible relationships among light dose, drug loading, mechanical properties, and release behavior. Therefore, although VAM offers a promising route toward high-speed fabrication of personalized drug products and drug-delivery devices, further advances in pharmaceutical-grade materials, formulation optimization, and quality-control strategies are required before broader clinical translation.

### 2.4. Matching Printing Technologies with Dosage-Form Functions

The suitability of a pharmaceutical 3D printing technology should not be evaluated solely based on resolution, printing speed, or geometric complexity. Instead, it should be determined by whether the material window, processing stress, post-processing requirements, and potential quality risks of the technology are compatible with the intended dosage-form function and release mechanism [52,53]. For oral printlets and programmable release systems, extrusion-based printing should be considered as a broad platform that includes both FDM and DIW/SSE. Although these approaches differ in material state and processing requirements, they share similar structural-programming capabilities because both rely on nozzle-based deposition along digitally defined paths. Accordingly, parameters such as infill density, shell thickness, pore architecture, channel design, multilayer organization, and spatial material distribution can be used in both systems to regulate water uptake, diffusion distance, exposed surface area, and release kinetics [54,55]. The main difference between FDM and DIW/SSE lies not in the types of structures that can be produced, but in process-specific limitations associated with their respective material systems. FDM is mainly limited by thermal exposure, melt processability, filament or feedstock properties, and drug thermal stability [56,57], whereas DIW/SSE is mainly affected by ink rheology, solvent removal, drying shrinkage, residual solvent control, drug migration, and post-printing structural retention [33,34].

For immediate-release or orally disintegrating formulations, powder-bed printing and DIW/SSE are useful because they can promote rapid medium penetration, high porosity, and fast disintegration. In contrast, FDM typically requires additional design strategies, such as hydrophilic matrices, low infill density, internal channels, disintegrants, or pore-forming excipients, to achieve accelerated drug release. For sustained-release, delayed-release, gastrointestinal-targeted, and multi-drug formulations, extrusion-based printing plays a particularly important role because shell structures, barrier layers, internal channels, and compartmentalized architectures can be directly incorporated into the dosage form during printing [55]. SLS may also contribute to these applications through porosity regulation and sintering-induced solid-state modification, whereas BJ is more commonly associated with highly porous, rapid-disintegration systems [58]. Compared with these technologies, photopolymerization-based printing is more relevant to microneedles, ocular inserts, hydrogel devices, and localized implants than to high-dose oral printlets. This formulation-driven selection principle also provides a basis for polymer selection, as polymers must simultaneously meet the requirements of processability, structural retention, drug compatibility, and release regulation [52,54]. The processing characteristics, pharmaceutical applications, and formulation constraints of the major 3D printing technologies are summarized in Table 1.

**Table 1:** Representative processing characteristics, pharmaceutical applications, and formulation constraints of major 3D-printing technologies.

Technology	Typical Processing Conditions and Resolution	Material	Suitable Formulation	State of the Printed Object	Advantages	Quality Risks	Translational Concerns	Study
FDM	~150–250 °C; layer height ~100–300 μm	Thermoplastic filaments; PVA, HPC, HPMC, Eudragit, Soluplus, PCL, PLA, TPU	Controlled-release printlets, gastroretentive systems, polypills	Thermoplastic polymer filaments	Structurally robust, mature equipment, suitable for complex internal structures	Heat exposure, filament brittleness, feeding instability, limited drug-loading capacity	Filament preparation, thermal process control, batch-to-batch consistency	[16,26,29]
DIW	Ambient to moderately heated; nozzle ~0.4–0.8 mm, layer height ~0.4–1.2 mm	Gels, slurries, and semi-solid inks; HPMC, PVP, PVA, Poloxamer, gelatin, alginate	Orodispersible formulations, pediatric formulations, chewable or semi-solid oral dosage forms, high-drug-loading formulations	Gels, pastes	Low-temperature processing, high formulation flexibility, suitable for heat-sensitive drugs	Rheological instability, drying shrinkage, poor dimensional stability	Ink standardization, post-printing drying/curing control, shape fidelity	[28,33,59]
BJ	Near ambient; post-drying often ~40–60 °C; layer height ~150–250 μm	Powder beds with binders; solid powders, PVP/HPC binders, sugar alcohols	Rapid-disintegrating oral formulations, high-dose immediate-release formulations	Powder blends and binder solutions	High porosity, rapid dispersion, and commercial precedent	Low mechanical strength, difficulty in powder removal and de-dusting, uneven binder distribution	Powder recovery, dust control, mechanical robustness, scale-up feasibility	[36,38,39]
SLS	Powder bed ~75–125 °C; layer height ~100–150 μm	Polymer powders; PEO, Eudragit, EC, PCL, PLA, Kollicoat	Porous printlets, immediate-release and sustained-release formulations	Thermoplastic polymer powders or powder formulations	Solvent-free processing, support-free fabrication, good structural integrity	Heat input, variability in powder spreading, risks from light absorbers	Powder handling, laser parameter optimization, safety evaluation of absorbers	[23,40,58]
SLA	Room-temperature photopolymerization; typical SLA Z-resolution ~10–200 μm	Photocrosslinkable resins or hydrogel precursors; PEGDA, GelMA, photocrosslinkable hydrogels	Microneedles, implants, punctal plugs, local drug delivery devices	Photocurable materials, primarily photosensitive resins	High resolution, good surface quality, suitable for microstructures	Photopolymerization residues, unreacted groups, incomplete curing	Safety evaluation, residual control, limited availability of pharmaceutically acceptable photocrosslinkable materials	[41,42,45]

Note: BJ, binder jetting; DIW, direct ink writing; EC, ethyl cellulose; FDM, fused deposition modeling; GelMA, gelatin methacryloyl; HPC, hydroxypropyl cellulose; HPMC, hydroxypropyl methylcellulose; PCL, polycaprolactone; PEGDA, poly(ethylene glycol) diacrylate; PEO, polyethylene oxide; PLA, polylactic acid; PVA, polyvinyl alcohol; PVP, polyvinylpyrrolidone; SLA, stereolithography; SLS, selective laser sintering; TPU, thermoplastic polyurethane.

## 3. Polymer Materials in Pharmaceutical 3D Printing: From Printability to Drug Delivery Functions

### 3.1. The Multiple Roles of Polymers in Pharmaceutical 3D Printing

In printed dosage forms, polymers serve functions beyond simply acting as carriers for active ingredients [15,60]. They must simultaneously enable processability within a suitable printing window, maintain structural integrity after deposition or solidification, facilitate drug dispersion or stabilization, and regulate wetting, hydration, dissolution, diffusion, erosion, or degradation processes during release [29]. Therefore, polymer selection should not rely only on chemical classification or conventional excipient functions, but should also consider material behavior during printing and its contribution to the final release mechanism. Since different printing technologies impose distinct material requirements, the same polymer may exhibit different pharmaceutical functions depending on the printing process. To avoid redundancy with the process-specific requirements discussed above, this section focuses on the functional roles of polymers and excipients in drug delivery. From a formulation perspective, commonly used materials can be categorized into rapidly dissolving polymers and release-enhancing excipients, hydration-controlled gel-forming polymers, pH-responsive polymers for site-specific release, non-erodible diffusion-barrier polymers, slowly degradable structural polymers, and photocrosslinkable polymers or hydrogel networks [61,62]. Importantly, polymer functions are not determined by material properties alone but are also expressed through the printed architecture [63,64]. The same polymer may promote rapid drug release when incorporated into a porous structure, while enabling sustained release when used in dense, thick-shell, or compartmentalized architectures. Not all polymers reported in 3D drug printing research possess an established history of use as oral pharmaceutical excipients. Materials such as PLA, TPU, and ABS have been investigated for their favorable thermoplasticity, mechanical properties, or structural stability; however, their status in pharmaceutical applications differs significantly from that of established excipients like HPMC, HPC, PVA, PVP, and pharmaceutical-grade methacrylate copolymers. While PLA is widely used in the biomedical field—particularly for biodegradable implants and drug delivery devices—its application as a conventional oral excipient remains very limited. TPU is primarily explored for specialized drug delivery devices and experimental printed dosage forms due to its excellent mechanical properties. ABS is essentially an engineering thermoplastic and should be regarded as a material for research or proof-of-concept purposes rather than as an established oral pharmaceutical excipient. Therefore, although studies utilizing these materials demonstrate manufacturing or structural feasibility, this should not be interpreted as direct evidence that these materials meet regulatory requirements for oral pharmaceuticals. Therefore, the critical consideration is not simply whether a polymer is printable, but whether its processing characteristics and release-regulating functions can be integrated with structural design to achieve predictable pharmaceutical performance [60].

In addition to the primary polymer matrix, functional excipients play an important role in determining both printability and drug-release performance, particularly in FDM-based formulations. Plasticizers such as polyethylene glycol, triethyl citrate, glycerol, and selected surfactants can reduce the glass-transition temperature and melt viscosity of polymeric matrices, thereby improving filament flexibility, feedability, and extrusion behavior. Fillers can modify mechanical strength, dimensional stability, and drug-loading capacity, whereas disintegrants such as crospovidone and croscarmellose sodium promote water penetration and tablet breakup to compensate for the relatively dense structure of FDM printlets. Porogens and water-soluble additives can further increase internal porosity and facilitate medium penetration, thereby accelerating drug release. Importantly, the functional role of an excipient in 3D printing may differ from that in conventional manufacturing; for example, additives traditionally used as lubricants or fillers may also act as plasticizers or rheology modifiers during thermal extrusion. Therefore, excipient selection should consider both conventional pharmaceutical functionality and its effects on processability, filament mechanics, structural integrity, and drug release during additive manufacturing. The pharmaceutical applicability of printable polymers should also be distinguished from their technical printability. Materials already used as inactive ingredients in approved drug products have a different regulatory starting point from polymers adopted primarily from engineering or biomedical additive manufacturing. The FDA Inactive Ingredient Database (IID), for example, provides route- and dosage-form-specific information on inactive ingredients present in approved drug products and can be used as supporting evidence during excipient selection. However, inclusion of a material in pharmaceutical 3D-printing studies does not itself establish its suitability for oral administration. Therefore, polymers should be evaluated not only for printability and release-regulating performance but also for prior pharmaceutical use, route-specific excipient status, residual impurities, degradation products, and the level of safety evidence required for the intended dosage form.

### 3.2. Rapid-Dissolving and Release-Enhancing Polymers

Rapidly dissolving polymers and release-enhancing excipients are primarily used in immediate-release and orally disintegrating formulations, as well as in systems developed to improve the dissolution of poorly soluble drugs [61]. Their main role is to promote medium penetration, matrix wetting, disintegration, drug dispersion, and dissolution; however, the extent of their release-enhancing effects is influenced by polymer viscosity, drug solubility, porosity, and the architecture of the printed dosage form [65,66]. Representative materials include hydrophilic thermoplastic or film-forming polymers such as PVA, PVP, Kollidon VA64, selected low-viscosity HPMC/HPC grades, and amphiphilic solubilizing carriers such as Soluplus. In addition, release-enhancing excipients, such as sugar alcohols, cyclodextrin derivatives, pore-forming agents, and disintegrants, are frequently incorporated to further improve drug dissolution and dispersion [67,68]. In FDM, hydrophilic thermoplastic carriers such as PVA and Kollidon VA64 can reduce diffusion barriers within the polymer matrix and promote hydration-driven drug release. Meanwhile, Kollidon VA64 and Soluplus can facilitate the stabilization of amorphous solid dispersions and improve the dissolution performance of poorly soluble drugs [65,69]. In DIW/SSE and powder-bed printing, rapidly dissolving materials can synergize with porous or low-density architectures to reduce wetting time and shorten diffusion pathways [70,71]. Therefore, the role of these polymers in pharmaceutical 3D printing is not determined solely by their intrinsic dissolution properties, but rather by the combined effects of material dissolution, pore structure, exposed surface area, and printed pathways on mass transfer regulation [61,66]. However, the use of these materials also presents formulation and quality challenges. High moisture sensitivity, excessive hydrophilicity, or rapid excipient dissolution may negatively affect storage stability, mechanical strength, filament feeding, dimensional stability, and dissolution reproducibility. In high-drug-load formulations, drug particles may interfere with the formation of a continuous polymer phase, leading to extrusion instability, heterogeneous wetting, reduced matrix integrity, and non-uniform drug release. Therefore, rapidly dissolving polymers and release-enhancing excipients should be evaluated in combination with drug solubility, process-specific formulation strategies, printed porosity, mechanical robustness, and storage stability, rather than being selected solely based on their hydrophilic properties [61].

### 3.3. Hydration-Controlled Gel-Forming Polymers

Hydration-controlled gel-forming polymers absorb aqueous media and generate hydrated gel layers that regulate drug release through swelling, polymer relaxation, diffusion, and matrix erosion or dissolution processes [72,73]. HPMC, HPC, PEO, and selected cellulose derivatives are widely used representatives of this material class [74]. Unlike rapidly dissolving polymers, the primary function of gel-forming polymers is not to promote immediate disintegration, but to establish a dynamic hydrated matrix that controls water penetration and drug diffusion [72,73]. In pharmaceutical 3D printing, these polymers are commonly incorporated into sustained-release scaffolds, gastroretentive systems, core–shell structures, and multilayer printlets [75,76]. Their release performance is determined by multiple factors, including polymer viscosity grade, molecular weight, hydration capacity, swelling and erosion behavior, drug solubility, and the architecture of the printed dosage form [74,77]. High-viscosity HPMC-based materials generally form stronger and more persistent gel layers, whereas lower-viscosity grades may promote faster hydration, matrix relaxation, or erosion, especially when combined with porous structures or release-enhancing excipients [74]. Beyond intrinsic polymer properties, printed architectures further modulate gel-controlled release by modifying porosity, shell thickness, open channels, and diffusion pathways [78,79]. Therefore, gel-forming polymers provide a useful example of how material-controlled hydration can be integrated with structure-defined diffusion pathways [73,76]. Compared with conventional hydrophilic matrices, 3D-printed systems introduce additional structural variables, including infill patterns, shells, channels, and internal cavities, which can alter hydration fronts, gel-layer formation, medium penetration, and drug diffusion routes [78]. Therefore, gel-forming polymers should be considered not merely as sustained-release excipients, but as functional matrices that can be combined with digital structural design to construct programmable release systems [59,80]. However, the major challenge associated with these materials is the dynamic and sometimes unpredictable evolution of swelling, erosion, and structural changes during dissolution. Excessive swelling may lead to deformation, channel blockage, or deviation from the intended release profile, whereas excessive erosion or dissolution may compromise scaffold integrity [81]. For DIW/SSE systems, water- or solvent-based inks may additionally experience drying shrinkage, cracking, or drug migration during post-printing processing [74,82]. Therefore, gel-forming polymer systems should be evaluated not only based on their initial formulation properties, but also according to pre-printing rheological behavior, post-printing dimensional stability, and dynamic structural evolution during dissolution and release testing [80].

### 3.4. pH-Responsive and Site-Specific Release Polymers

pH-responsive polymers can dissolve, hydrate, or alter their permeability in response to gastrointestinal pH conditions, making them suitable for site-specific gastric, enteric, and distal intestinal delivery systems [83,84]. Representative materials include Eudragit E, Eudragit L, Eudragit S, Eudragit FS, HPMCAS, and CAP [85]. The key characteristic of these polymers is not simply their solubility, but rather the compatibility between their pH-response thresholds and the intended release environment [86]. For example, Eudragit E remains largely insoluble at salivary pH but dissolves under acidic gastric conditions, making it suitable for taste masking and gastric release applications. In contrast, Eudragit L-type polymers, HPMCAS, and CAP are mainly used for enteric or small-intestinal release, whereas Eudragit S100 and FS-type polymers are more frequently investigated for distal intestinal or colon-targeted delivery [84,85]. In 3D-printed formulations, pH-responsive polymers can serve functions beyond conventional coating materials. They can be spatially incorporated as shells, barrier layers, separation layers, matrix domains, or functional compartments within multi-material architectures [11,87]. This structural integration enables pH-responsive regions to be combined with drug-loaded domains, scaffold structures, pores, or diffusion barriers, thereby facilitating delayed, sequential, or compartmentalized drug release [88]. In multi-drug formulations, spatial organization using pH-responsive materials may further reduce drug incompatibility and enable differentiated release profiles [86,89]. However, the application of pH-responsive polymers in pharmaceutical 3D printing faces challenges related to processing adaptability, preservation of pH-response thresholds, and in vivo relevance. In FDM, these polymers may require plasticization or blending strategies to overcome filament brittleness, high melt viscosity, thermal stress, or drug-polymer incompatibility during processing [90,91]. In DIW/SSE, dispersion stability, solvent compatibility, drying shrinkage, residual solvent control, and structural retention require careful optimization [82]. More importantly, drug release triggered by pH changes in compendial dissolution media may not fully reflect in vivo performance, as gastrointestinal pH, transit time, food intake, disease conditions, and interindividual variability can influence both release onset and release extent. Therefore, pH-responsive polymers in printed formulations should be evaluated through an integrated assessment of in vitro release behavior, structural integrity, process robustness, and in vivo relevance [84,92].

### 3.5. Non-Erodible Diffusion-Barrier and Slowly Degradable Polymers

Non-erodible diffusion-barrier polymers and slowly degradable structural polymers are particularly useful for formulations requiring long-term structural integrity, extended diffusion pathways, or localized sustained release [93,94]. EC, CA, EVA, and TPU are commonly employed as diffusion-barrier or non-erodible matrix materials, whereas PCL, PLA, and PLGA are more frequently used as slowly degradable structural polymers [95]. Although both material groups can maintain structural integrity during drug release, their underlying mechanisms differ. Non-erodible diffusion-barrier polymers mainly regulate drug release by restricting wetting pathways, controlling membrane permeability, and limiting drug diffusion, whereas slowly degradable structural polymers provide long-term mechanical support and regulate release through diffusion, polymer degradation, or the combination of both mechanisms [96]. In extrusion-based printing, thermoplastic polymers such as PLA, PCL, TPU, and EVA have been investigated for implants, support frameworks, gastroretentive structures, and sustained-release printlets due to their mechanical strength and structural stability [93,95]. EC and CA, in contrast, primarily function as diffusion barriers and can be incorporated into sustained-release or pulsatile-release systems by reducing water ingress, modifying membrane permeability, or working synergistically with porogens [97,98]. For implantable or long-acting local delivery systems, PCL and PLGA can further regulate drug release through polymer degradation, drug diffusion, and drug-polymer interactions [94,96]. The major advantage of these materials in 3D printing lies in their ability to maintain geometric integrity during the release process, thereby making parameters such as shell thickness, pore size, surface area-to-volume ratio, and diffusion path length important determinants of release kinetics [64,99]. However, excessive diffusion restriction may result in incomplete drug release, high residual drug levels, or insufficient initial release unless hydrophilic components, porogens, or rational pore structures are incorporated. Thermoplastic materials such as PLA, PCL, TPU, and EVA often require elevated processing temperatures, which may limit their application in formulations containing heat-sensitive drugs [93]. For degradable polymers, degradation products, local pH variations, long-term biocompatibility, and the predictability of in vivo release profiles require further investigation. Therefore, these materials should be considered primarily as structural and diffusion-controlling components for sustained-release, implantable, or localized delivery systems, rather than direct substitutes for rapidly dissolving immediate-release matrices [94].

### 3.6. Photocrosslinkable and Hydrogel Polymers

Photocrosslinkable polymers and hydrogel networks can form crosslinked matrices with tunable mesh size, hydration behavior, mechanical properties, and drug diffusion characteristics. Representative materials include PEGDA, PEGDMA, GelMA, methacrylated polysaccharides, and other photocrosslinkable hydrogel precursors [41,42,62]. The release-regulating functions of these materials are mainly determined by network characteristics, including crosslinking density, pore size, hydrophilicity, degradability, and drug-network interactions. By adjusting monomer concentration, crosslinker ratio, irradiation conditions, and post-curing parameters, the diffusion of drugs through the crosslinked matrix can be precisely modulated [24,100]. In addition to the polymer matrix, photoinitiator type and concentration are important formulation variables. Irgacure 2959 is commonly used in photocrosslinkable hydrogel systems because of its relatively low cytotoxicity compared with several conventional photoinitiators, although its limited absorption at longer wavelengths may require prolonged irradiation. Lithium phenyl-2,4,6-trimethylbenzoylphosphinate (LAP) provides more efficient initiation under near-UV or visible-light conditions and can reduce the exposure time required for crosslinking. However, the cytotoxicity of both photoinitiators is concentration- and irradiation-dependent, as increasing photoinitiator concentration can enhance curing efficiency while also increasing radical generation and exposure to residual photoproducts. Tartrazine is commonly incorporated as a photoabsorber rather than a radical-generating photoinitiator; by restricting excessive light penetration, it can improve curing resolution, although excessive concentrations may reduce curing depth and result in incomplete crosslinking. Therefore, photoinitiator and photoabsorber concentrations must be optimized together with irradiation wavelength, intensity, and exposure time to balance curing efficiency, structural fidelity, and biocompatibility.

At present, these materials remain more suitable for microstructured or localized delivery systems than for high-dose oral printlets. Their pharmaceutical applications are primarily associated with microneedles, ocular inserts, hydrogel devices, localized implants, and drug-eluting systems. However, their clinical translation remains limited by several challenges, including unreacted monomers, residual photoinitiators, incomplete curing, crosslinking side reactions, light-induced drug instability, limited drug-loading capacity, and regulatory uncertainty [42,45,101]. Incomplete polymerization is particularly important because residual acrylate or methacrylate monomers, oligomers, photoinitiators, and photolysis products may leach from the printed matrix and contribute to concentration-dependent cytotoxicity. Post-printing washing and additional curing can reduce these residual components, but excessive washing may remove soluble drugs or excipients, whereas additional light exposure may further alter crosslinking density and drug-release behavior. Moreover, APIs may undergo direct photodegradation, radical-mediated oxidation, or drug-polymer reactions during SLA/DLP processing, depending on their intrinsic photostability and the applied wavelength and light dose. Therefore, photocrosslinkable and hydrogel materials should be considered specialized material platforms for microstructured and localized drug delivery, rather than general-purpose polymers for oral 3D-printed formulations. Future studies should additionally evaluate monomer conversion, residual extractables and leachables, photoinitiator concentration, and API photostability under the actual irradiation and post-curing conditions used during manufacturing.

### 3.7. Linking Polymer Functions, Printing Processes, and Structural Release Control

In printed dosage forms, release kinetics are not determined solely by polymer chemistry, but rather arise from the combined effects of material properties, printing processes, and spatial architecture [5]. Polymer characteristics, including hydrophilicity, molecular weight, hydration capacity, gel formation, pH responsiveness, and degradation rate, define the fundamental mechanisms governing drug release. Printing processes determine whether the formulation can be manufactured reproducibly. Meanwhile, design parameters such as infill density, porosity, shell thickness, pore size, surface area-to-volume ratio, and material distribution further regulate medium penetration pathways, diffusion distances, and drug spatial organization [63,64]. This process-structure-release relationship represents a key distinction between 3D-printed formulations and conventional dosage forms. Traditional formulations typically regulate release through composition, compaction pressure, coating thickness, or granulation processes, whereas 3D printing enables structural parameters to be directly incorporated into the dosage form design [64]. A single hydrophilic polymer may promote rapid release when incorporated into a highly porous structure but achieve sustained release when used in a high-infill, thick-shell, or core–shell architecture. Similarly, a hydrophobic polymer may serve different functions, including a sustained-release scaffold, an external diffusion barrier, or a compartmentalization material, depending on its spatial arrangement within the printed structure [63,66,86]. Therefore, polymer materials alone do not determine formulation functionality; rather, the key factor is the compatibility between material properties and printed architectures. Future research should move beyond simply determining whether a particular polymer is printable and instead establish quantitative relationships among material properties, process parameters, structural variables, and release behavior. Accordingly, the central challenge in pharmaceutical 3D printing is not simply to identify additional printable excipients, but to develop formulation-oriented principles that integrate material selection, processability, structural design, and release performance. Only when polymer processability, structural retention, and release-regulating performance become predictable and reproducible can printed formulations advance from case-specific demonstrations toward scalable and reliable drug delivery systems. The functional classifications, representative materials, compatible printing technologies, and major challenges of polymers and functional excipients used in pharmaceutical 3D printing are summarized in Table 2. Representative studies illustrating the relationships among printing methods, polymer matrices, model drugs, dosage-form designs, and experimentally observed drug-release behaviors are summarized in Table 3.

**Table 2:** Polymers and functional excipients used in pharmaceutical 3D printing.

Functional Classification	Representative Materials	Primary Compatible Technologies	Key Material Functions	Typical Dosage Forms	Major Challenges
Rapid-dissolving/ Fast-release	PVA, PVP, Kollidon VA64, Low-viscosity HPMC/HPC grades, Sugar alcohols, Cyclodextrins	FDM, DIW/SSE, SLS, BJ	Rapid wetting, Disintegration, Solubilization, Improved dissolution	Orally disintegrating, Immediate-release, Highly porous printlets	Hygroscopicity, Low mechanical strength, Poor storage stability
Swelling-dissolution polymers	HPMC, HPC, PEO, Cellulose derivatives	FDM, DIW/SSE	Gel-layer formation, Hydration barrier, Diffusion retardation	Controlled release, Gastric retention, Core–shell structure	Swelling deformation, Variable erosion rate
pH-responsive polymers	Eudragit E/L/S/FS, HPMCAS, CAP	FDM, DIW/SSE, SLS	Gastric dissolution, Enteric protection, Colonic-target release	Enteric, Colon-targeted, Multi-stage release	Processing brittleness, pH interindividual variability
Non-erosion barrier-forming polymers	EC, CA, EVA, TPU	FDM, DIW/SSE	Reduced medium penetration, Extended diffusion pathways	Sustained-release, Coated, Pulsed-release	Overly slow drug release, Dependence on porogens
Slow-degrading polymers	PCL, PLA, PLGA	FDM, SLS, In situ printing	Structural support, Sustained release, Localized delivery	Implants, Scaffolds, Localized drug delivery	High-temperature processing, Long-term safety
Photocrosslinkable hydrogel-forming polymers	PEGDA, GelMA, photocrosslinkable polysaccharide derivatives	SLA, DLP, CLIP, TPP	Cross-linked networks, Microstructure formation	Microneedles, Lacrimal Plugs, Hydrogel devices	Photoinitiator residue, Unreacted functional groups

Note: BJ, binder jetting; CA, cellulose acetate; CAP, cellulose acetate phthalate; CLIP, continuous liquid interface production; DIW, direct ink writing; DLP, digital light processing; EC, ethyl cellulose; EVA, ethylene-vinyl acetate copolymer; FDM, fused deposition modeling; GelMA, gelatin methacryloyl; HPC, hydroxypropyl cellulose; HPMC, hydroxypropyl methylcellulose; HPMCAS, hydroxypropyl methylcellulose acetate succinate; PCL, polycaprolactone; PEGDA, poly(ethylene glycol) diacrylate; PEO, polyethylene oxide; PLA, polylactic acid; PLGA, poly(lactic-co-glycolic acid); PVA, polyvinyl alcohol; PVP, polyvinylpyrrolidone; SLA, stereolithography; SLS, selective laser sintering; SSE, semi-solid extrusion; TPP, two-photon polymerization; TPU, thermoplastic polyurethane.

**Table 3:** Summary of printing methods, polymers, model drugs, dosage forms, and release behaviors in 3D-printed pharmaceutical formulations.

Printing Method	Polymer Matrix	Model Drug(s)	Dosage Form	Release Behavior	Study
FDM	PEO	Gabapentin	Gastroretentive printlet	Extended release; floating for >10 h in the optimized two-shell, 0% infill formulation	[75]
FDM	HPMCAS	Paracetamol	Enteric printlet	Delayed release (<10% release within the first 2 h; complete release at ~8 h; slowest release >24 h)	[102]
FDM	Eudragit EPO, Soluplus, PVA	Felodipine	Disc-shaped solid dispersion printlet	Sustained and zero-order release	[103]
FDM	Eudragit RL, RS, E; HPC	Theophylline	Printlet	Immediate- and extended-release profiles (IR within 25 min; ER up to 16 h)	[104]
FDM	PVA	Paracetamol; caffeine	Multilayer core–shell printlet	Sustained release	[105]
FDM	PVA	Paracetamol; caffeine	Printed caplet	Sustained release (100% release within 8 h)	[65]
FDM	Eudragit L100-55, S100; PLA	5-Fluorouracil	Colon-targeted delivery system	pH-responsive colon-targeted release (controlled release at pH 7.4)	[81]
FDM	PVA	Ketoprofen	Channeled printlet	pH-dependent release (~90% release at 90 min and 100% release at 3 h)	[106]
FDM	PLA, PVA, HPMC, HPMCAS	Acetaminophen	Capsule	Two-pulse release (immediate and delayed pulses)	[88]
FDM	HPMC, Soluplus	Acetaminophen	Core–shell printlet	Zero-order sustained release	[73]
FDM	HPC, EC	Theophylline	Core–shell gastroretentive printlet	Floating pulsatile release (lag time: 30 min–6 h)	[30]
FDM	HPC, Eudragit RL PO, PEG	Theophylline	Printed caplet	Sustained release (~80% release within 10 h; 100% release at ~ 10 h)	[107]
FDM	PVA	Paracetamol	Printlet	Geometry-dependent controlled release	[8]
FDM	Eudragit E	Hydrochlorothiazide	Printed caplet	Channel-assisted release	[66]
FDM	HPC	Theophylline	Gaplet tablet	Immediate release by fragmentation (disintegration within 4 min; 86.7% release at 30 min)	[108]
FDM	PLA, PVA	Lisinopril; amlodipine; indapamide; rosuvastatin	Polypill capsules	Bespoke multi-drug release (>80% release at 30 min; 89% and 55% release at 24 h)	[31]
FDM and IVF	TPU, DrugCoat S 12	Theophylline	Colon-targeted system	Zero-order colon-specific release	[109]
FDM	PVA	Fluorescein	Printlet	Immediate-to-sustained release	[21]
FDM	PVA, CA, PVP	Diltiazem	Core–shell printlet	Osmotic-controlled release (onset: 0–360 min)	[110]
FDM	PVA	Ketoprofen	Multilayer printlet	Immediate and sustained release	[111]
FDM	HPMC	Metformin	Gastroretentive printlet	Gastroretentive sustained release (>8 h)	[112]
FDM	HPMCAS, PVA, Eudragit RL	Naringenin	Printlet	Nanoparticle-mediated release	[113]
FDM	PVA	Lisinopril; indapamide; rosuvastatin; amlodipine	Multilayer printlet	Architecture-dependent release	[114]
FDM	HPMCAS, PEG	Indomethacin	Chewable printlet	Immediate release (within 60 min)	[115]
FDM	PETOx, PLA	Dextromethorphan; hydrochlorothiazide	Printlet	Shell-dependent release (>80% release within 60 min for 1-shell; >60% release within 60 min for 5-shell)	[116]
FDM	HPC, PEO, HEC, ABS	Paracetamol	Core–shell printlet	Zero-order sustained release (12–14 h)	[117]
FDM	PVA, Eudragit L100	Budesonide	Printed caplet	Enteric-controlled release (up to 24 h)	[118]
FDM	PLA, PVA, PCL	Methylene blue; ibuprofen sodium; ibuprofen acid	Implantable construct	Prolonged implant release	[119]
FDM	Eudragit EPO, PEO	Zolpidem hemitartrate	Printed caplet	Size- and drug-load-dependent release (>80% release at ~45–60 min for small caplets and >200 min for large caplets)	[120]
FDM	PVA, EVA	Pramipexole; levodopa; benserazide	Gastroretentive polypill	Floating sustained release (75% release at 720–750 min; 100% release at 1440 min)	[121]
FDM	PVA	Paracetamol	Core–shell printlet	Geometry-dependent release	[122]
APF-DDM, FDM	PLGA	Ibuprofen	Mesh-shaped implantable construct	Prolonged implant release	[123]
FDM	PVA, EVA	Pramipexole; levodopa; praziquantel	Printlet	SA/V-governed release	[124]
FDM	HPMCAS, HPMC E4, PEG, PVA	Pregabalin	Gastroretentive printlet	Intragastric floating controlled release	[125]
FDM	HPMCAS, PVA	5-ASA	Bicompartmental colon-targeted device	pH-dependent colon-specific release	[126]
FDM	PCL	Ibuprofen	3D construct	Porosity-controlled release	[127]
FDM	HPMCAS, HPC-EF	Carbamazepine	Printlet	Printing-parameter-dependent release	[128]
FDM	HP-60D-35, ATPU-75A, PLGA, HPMC	Hydroxychloroquine; IgG; gp120; C6NP	Intravaginal ring	Multiple-drug intravaginal ring release	[129]
FDM	PVA, PLA, TPU	Nifedipine	Multilayer printlet	Sustained release	[130]
FDM	PVA, PVP	Itraconazole	Printlet	Immediate release	[67]
FDM	PVA, TPE-E	Allopurinol	Gastroretentive printlet	Floating gastric controlled release	[131]
FDM	PLA, PVOH	Fenofibrate; halofantrine; lumefantrine; clofazimine	Polymer-lipid hybrid printlet	SMEDDS-enabled rapid self-emulsifying release	[132]
FDM	HPC, HPMC, PEO, Eudragit	Isoniazid	Printlet	Dose-adjustable release	[133]
FDM	HPC, HPMCAS	Melatonin; caffeine	Modular LEGO-like printlet	LEGO-like modular tailored release profiles	[37]
FDM	HPC, Soluplus	Curcumin; lansoprazole	Hollow printlet	Compartmentalized bio-SNEDDS release	[134]
FDM	PVA, Kollidon SR	Diclofenac sodium	Bilayer printlet	Bilayer immediate- and sustained-release profile	[135]
FDM and IVF	PLA, HPMC, Eudragit FS30D	Theophylline	Colon-targeted system	Printfill-based release	[136]
FFF, DEAM	EVA	Metoprolol tartrate	Printlet	Feedstock-dependent release	[137]
FFF	HPMCAS, PEG, PVA	Acetaminophen	Enteric capsule	Customizable enteric capsule release	[87]
DIW	Cellulose acetate, HPMC, PVP K30	Aspirin; hydrochlorothiazide; pravastatin; atenolol; ramipril	Multilayer system	Immediate and sustained release	[3]
DIW	HPMC	Indomethacin	Orodispersible film	Fast oral-film disintegration (approximately 1–2.5 min) and indomethacin release within approximately 2–3 min.	[138]
DIW	CMCS, SA, PEGDA	Bovine serum albumin/model bovine serum protein	Hydrogel printlet	pH-responsive polypill release	[139]
DIW	Gelatin, PVP, PVA	Propranolol HCl	Buccal film	Prolonged buccal release	[140]
DIW	Cellulose acetate	Propranolol HCl	Core–shell printlet	Coating-shell controlled release	[10]
DIW	Poloxamer 188, PEG 6000	Dapagliflozin	Self-nanoemulsifying printlet	Immediate release (>75% within 20 min)	[141]
DIW	HPMC, PVP	Tranexamic acid; paracetamol	Printlet	Pore-structure-dependent release	[33]
DIW	EC, CA, PEG, PEO, Sodium alginate	Bezafibrate	Osmotic pump tablet	Capable of achieving zero-order release	[98]
DIW	CMC-Na, PVP K30, CMS-Na	Levetiracetam	rapid release tablets	Faster release behavior	[142]
DIW	PVP K90, Eudragit^®^ RS (PO), Eudragit^®^ RL (PO)	Metformin hydrochloride	Core–shell delivery system	Facilitates sustained release of high-dose water-soluble formulations for over 12 h	[59]
DIW	Kollicoat^®^ IR	Levetiracetam	Orodispersible tablet (round-shaped, diameter 10 mm)	Increasing layers decreased drug release rate; release was related to the SA/V ratio.	[143]
DIW	EC, HPMC, PVP, CA	Immediate-release tablets	Controlled release shell	Controlled release shell	[144]
DIW	Mannitol, Maltodextrins, Croscarmellose sodium, Tween 80^®^	Acetaminophen, Celecoxib	Printlet	Immediate release	[145]
DIW	Milk formula as the primary excipient	Paracetamol (PCT), Sodium valproate (SDVT), Ibuprofen (IBU), Carbamazepine (CBZ)	Paediatric chewable dosage forms	Milk matrix markedly enhanced CBZ release, modestly improved IBU, and maintained rapid, near-complete release of highly soluble PCT and SDVT.	[146]
DIW	HPMC 4500	Losartan potassium	Printlet	Increasing drug loading enhanced the initial release rate, reaching up to 63% in 45 min for 15% loading.	[147]
Extrusion-based	PVP	Paracetamol	Printlet	Geometry-dependent release	[34]
Binder jetting	Vinyl polymer, Soluplus, PEG	5-Fluorouracil	Printlet	Fast release of 5-fluorouracil printlet	[22]
Binder jetting	GalenIQ™ 721, Ludiflash^®^, Pharmaburst^®^ 500, Kollidon^®^ VA64, Aerosil^®^ 200, Kollidon^®^ 30	No model drug was mentioned	Orally Disintegrating Tablets	No drug release study was conducted	[148]
Binder jetting	Calcium sulfate hemihydrate	Rhodamine 6G	Printlet	The higher the ethanol ratio, the faster the drug release and the higher the cumulative release. At 24 h, the cumulative release was 80.69% for WE0-100 and 57.18% for WE25-75.	[149]
Binder jetting	MCC PH101, Lactose, Mannitol, PVP K30	Acetaminophen	Personalized orally disintegrating tablets	Rapid disintegration and rapid release: complete disintegration within 10 s, and almost complete release within 5 min for all tablet specifications.	[150]
SLS	Kollicoat IR, Eudragit L100-55	Paracetamol	Printlet	Immediate and pH-dependent release (100% release within 12 h)	[151]
SLS	Ethyl cellulose, Kollicoat IR	Paracetamol; ibuprofen	Miniprintlets	Multi-drug controlled release (immediate and prolonged release profiles)	[152]
SLS	PEO, Eudragit L, RL; EC	Paracetamol	Gyroid lattice, bilayer printlet	Customisable release	[23]
SLS	Kollidon^®^ VA 64, HPMCAS	Rosuvastatin calcium, Acetylsalicylic acid	Bilayer tablets	Slower release at higher laser intensity (denser core, higher hardness, lower friability).	[153]
SLS	Parteck^®^ MXP, Kollidon^®^ VA 64	Efavirenz	Dispersible printlets	Similar disintegration times were found for P500, K500 and K1000 (<230 s), with only partial disintegration for P1000	[154]
SLS	HPMC, Kollidon^®^ VA 64	Atomoxetine hydrochloride	Printlet	Ultra-rapid release (>85% in 15 min) at high laser speeds; slower disintegration and harder tablets at low speeds	[155]
SLS	HPMC, Kollidon^®^ CL-M, Kollicoat^®^	Lamivudine	Printlet	Fast-disintegrating (DT 2.8 ± 1.2–43.7 ± 2.7 s) and fast-dissolving	[156]
SLS	Kollicoat^®^ IR, HPMC	Lamivudine, Tenofovir disoproxil fumarate	Combination printlet	Completely (>85%) dissolved in 20 min	[157]
SLS	Kollicoat^®^ IR, Eudragit^®^ L-100-55, StarCap^®^ 1500	Dapsone	Printlet	Rapid and complete (>80% in 10 min)	[158]
SLS	PVA	Indomethacin	Multi-layered tablets	Biphasic release at pH 6.8: quick initial release followed by prolonged/slow release reaching plateau after several hours	[159]
SLS	Kollicoat^®^ IR, MAS, MCC	Lopinavir	Printlet	Disintegrant-dependent dissolution: 71.1 ± 5.7–99.3 ± 2.7% within 120 min	[160]
SLA	PEGDA, PEG 300	4-ASA; paracetamol	Printlet	Sustained release (100% release within 10 h)	[161]
SLA	PEGDA	Paracetamol; caffeine; naproxen; chloramphenicol; prednisolone; aspirin	Multilayer system	Multi-drug distinct release (paracetamol/caffeine/aspirin ~100% release at 20 h; chloramphenicol/naproxen >80% release at 20 h; prednisolone 45% release at 20 h without complete release)	[89]
SLA	PEGDA	Paracetamol	Printlet	Geometry-dependent release	[162]
SLA	PEGDA, PEO	Berberine	Nanocomposite printlet	pH-dependent sustained release	[163]
DLP	AETMA, PEGDA	Acetylsalicylic acid	Hydrogel implantable construct	Pulsatile pH-dependent release	[25]
Droplet deposition	PLGA	Ibuprofen	Mesh-shaped implantable construct	Filling-density -dependent release	[63]
HME and PAM	PVA, HPMC	Tacrolimus	Core–shell printlet	Extended release (86% release at 12 h; similar dissolution profile to the commercial capsule)	[76]
Microextrusion	Pluronic F-127	Metformin; glyburide; acarbose	Core–shell, multilayer, and gradient system	Temporal release programming	[164]
APF	Eudragit E PO, Soluplus	Paracetamol; felodipine	Core–shell, multilayer printlet	Simultaneous, delayed, and pulsatile release	[86]
Inkjet printing	PCL, PLA, PTMC	Trandolapril; pitavastatin	Implantable construct	Prolonged implant release (1 week to months)	[95]
Direct powder extrusion	PVA	Loperamide	Printlet	Immediate-to-sustained release	[165]

Note: 4-ASA, 4-aminosalicylic acid; 5-ASA, 5-aminosalicylic acid; ABS, acrylonitrile butadiene styrene; AETMA, [2-(acryloyloxy)ethyl]trimethylammonium chloride; APF, Arburg Plastic Freeforming; APF-DDM, Arburg Plastic Freeforming–Droplet Deposition Modeling; ATPU, aliphatic thermoplastic polyurethane; bio-SNEDDS, bioactive self-nanoemulsifying drug delivery system; C6NP, coumarin-6 nanoparticles; CA, cellulose acetate; CMCS, carboxymethyl chitosan; DEAM, direct extrusion additive manufacturing; DIW, direct ink writing; DLP, digital light processing; EC, ethyl cellulose; ER, extended release; EVA, ethylene-vinyl acetate copolymer; FDM, fused deposition modeling; FFF, fused filament fabrication; HEC, hydroxyethyl cellulose; HME, hot-melt extrusion; HPC, hydroxypropyl cellulose; HPMC, hydroxypropyl methylcellulose; HPMCAS, hydroxypropyl methylcellulose acetate succinate; IgG, immunoglobulin G; IR, immediate release; IVF, injection volume filling; PAM, pressure-assisted microsyringe; PCL, polycaprolactone; PEG, polyethylene glycol; PEGDA, poly(ethylene glycol) diacrylate; PEO, polyethylene oxide; PETOx, poly(2-ethyl-2-oxazoline); PLA, polylactic acid; PLGA, poly(lactic-co-glycolic acid); PTMC, poly(trimethylene carbonate); PVA/PVOH, polyvinyl alcohol; PVP, polyvinylpyrrolidone; SA, sodium alginate; SA/V, surface area-to-volume ratio; SLA, stereolithography; SLS, selective laser sintering; SMEDDS, self-microemulsifying drug delivery system; TPE-E, thermoplastic polyester elastomer; TPU, thermoplastic polyurethane.

## 4. Structure-Enabled 3D-Printed Dosage Forms: From Rapid Disintegration to Programmable Release

The pharmaceutical value of 3D printing is most clearly demonstrated when structural parameters are used as formulation variables. Rather than simply generating complex geometries, printed dosage forms can use porosity, infill density, shell thickness, channels, and material partitioning to regulate mass transfer processes and control release timing. Conventional dosage forms mainly modulate drug release through formulation composition, compaction pressure, coating thickness, or granulation processes. In contrast, 3D printing enables geometric features, infill density, pore architecture, shell thickness, material partitioning, and spatial drug distribution to be directly integrated into dosage-form design [10,139]. Importantly, 3D printing usually does not introduce completely new release mechanisms; instead, it reorganizes and spatially regulates established mechanisms, including wetting, diffusion, erosion, degradation, and staged release. Across immediate-release, sustained-release, gastrointestinal site-specific, multicomponent, and localized delivery systems, the underlying design principle is to control how the medium penetrates the formulation, how far drugs diffuse, which surface regions are exposed, and when different drug-containing domains become accessible for release [11,81,84].

### 4.1. Immediate-Release and Rapid-Disintegrating Oral Printlets

**Figure 2:**
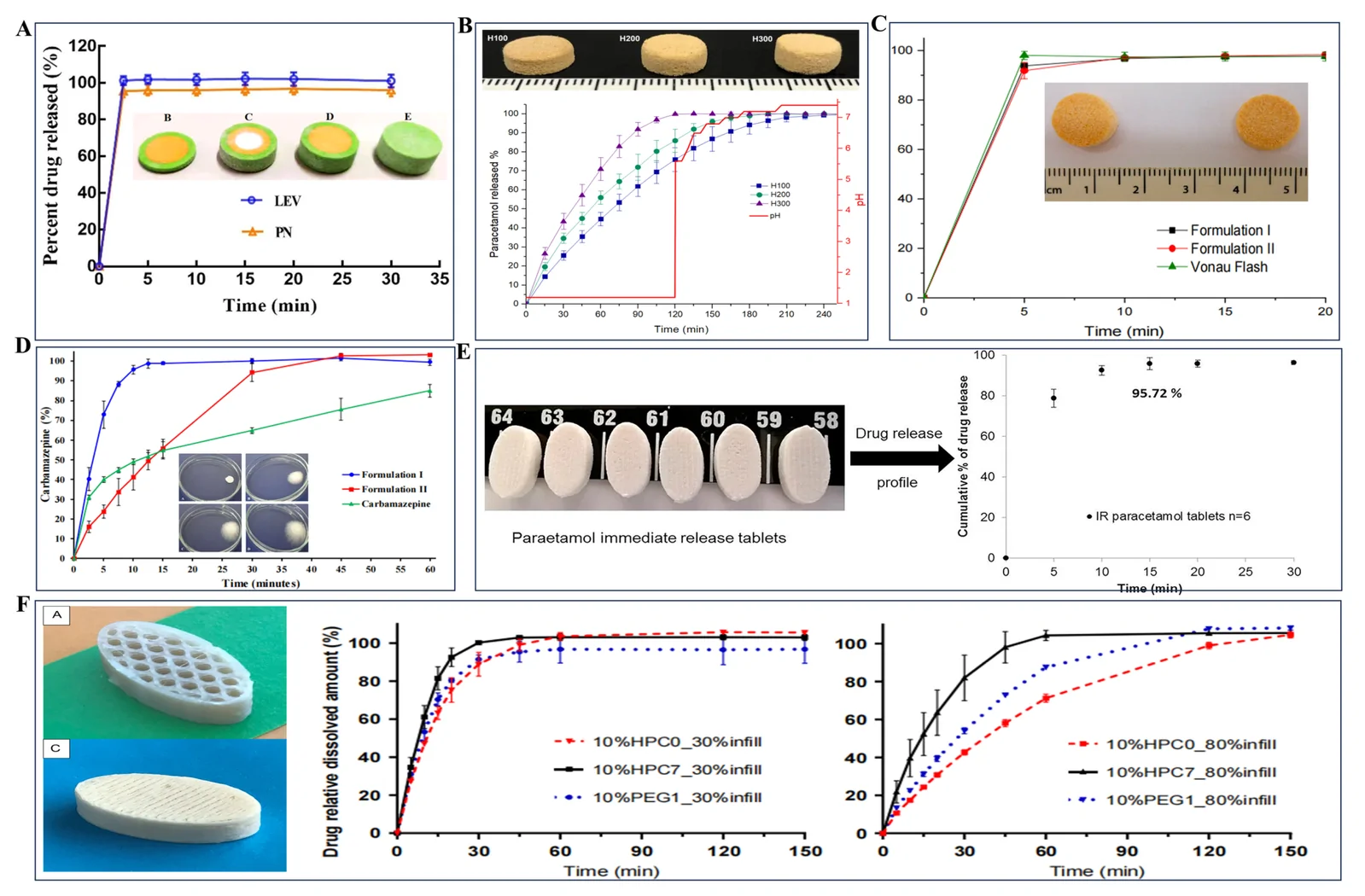
Representative 3D-printed immediate-release and orally disintegrating formulations. (**A**) Binder-jetting multichamber printlets containing a porous core and dense shell designed for rapid disintegration and improved mechanical stability [169]. (**B**) Low-energy SLS printlets balancing rapid wetting with structural integrity [170]. (**C**) SLS printlets integrating porous architectures with cyclodextrin-assisted solubilization strategies [70]. (**D**) Carbamazepine printlets containing HPβCD and cellulose derivatives for tunable rapid-release profiles [167]. (**E**) High-drug-load acetaminophen printlets fabricated by DIW/SSE using a hydrophilic paste containing disintegrants [171]. (**F**) Low-infill FDM printlets containing PEG 4000 and Kollidon VA64 for enhanced release acceleration [173]. Adapted with permission from the original publications.

For immediate-release and orally disintegrating printlets, structural design is primarily intended to facilitate aqueous medium penetration, matrix wetting, structural disintegration, and drug diffusion, rather than to prolong diffusion pathways or maintain long-term scaffold integrity [61,108]. Accordingly, the key design strategies include high porosity, low infill density, short diffusion pathways, hydrophilic or rapidly dissolving matrices, and approaches that promote dissolution or disintegration [166,167]. Representative 3D-printed immediate-release and orally disintegrating formulations employing these structural and formulation strategies are illustrated in Figure 2.

Powder-bed printing provides a clear demonstration of this principle. The commercialized Spritam^®^ formulation, produced using a powder-liquid 3D printing process, showed that highly porous printed matrices can rapidly absorb water and disperse, thereby helping to overcome swallowing difficulties associated with conventional high-dose oral tablets [168]. Building on this porous-matrix concept, Hong et al. further applied binder jetting to construct a multi-chamber dispersible formulation containing levetiracetam and pyridoxine hydrochloride, in which a loosely structured core promoted rapid disintegration while a denser shell maintained mechanical integrity [169]. This example illustrates that immediate-release structures should not simply maximize porosity; instead, they must balance rapid dispersion, mechanical robustness, and dose organization. SLS extends the powder-bed strategy by using laser energy to consolidate polymer powders while preserving tunable porosity. Fina et al. demonstrated that low-energy SLS conditions could produce rapidly dispersing orodispersible printlets, showing that powder sintering parameters can be adjusted to regulate the balance between structural integrity and rapid wetting [170]. Allahham et al. further incorporated drug-cyclodextrin solubilizing systems into SLS printlets, indicating that pore architecture can be combined with formulation-level solubilization strategies to improve the dissolution of poorly soluble drugs [70].

Extrusion-based printing offers another strategy for achieving immediate release, especially when mild processing conditions, high drug loading, or flexible small-batch manufacturing are required. Khaled et al. used a water-based paste containing PVP K25, cross-linked sodium carboxymethylcellulose, and starch to fabricate high-drug-load immediate-release acetaminophen printlets. The reported strategy demonstrated the potential of DIW/SSE to avoid thermal stress while promoting disintegration through the incorporation of hydrophilic and disintegrating components [34,171]. Conceição et al. further combined carbamazepine with HPβCD and cellulose derivatives, showing that cyclodextrin complexation, matrix composition, and printed porosity can be integrated to modulate release profiles ranging from orodispersible to immediate-release formulations [167].

FDM can also be adapted for immediate-release printlets, although its relatively dense thermoplastic matrices often restrict medium penetration. Therefore, FDM-based immediate-release formulations generally require compensatory strategies such as low infill density, open channels, hydrophilic carriers, disintegrants, pore-forming excipients, and release-promoting additives [61,172]. For example, Fanous et al. accelerated drug release by introducing low-infill, high-porosity structures and PEG4000/Kollidon VA64-based release-promoting systems [173]. Đuranović et al. further showed that combining Kollidon CL with mannitol effectively enhanced acetaminophen dissolution from FDM printlets [174], whereas Yilmaz et al. enhanced erosion and immediate-release behavior by incorporating MCC II and reducing packing density [175]. These studies confirm that immediate-release FDM systems are feasible; however, their performance relies more heavily on structural opening and formulation compensation compared with powder-bed and DIW/SSE systems.

### 4.2. Sustained-Release and Controlled-Release Formulations

**Figure 3:**
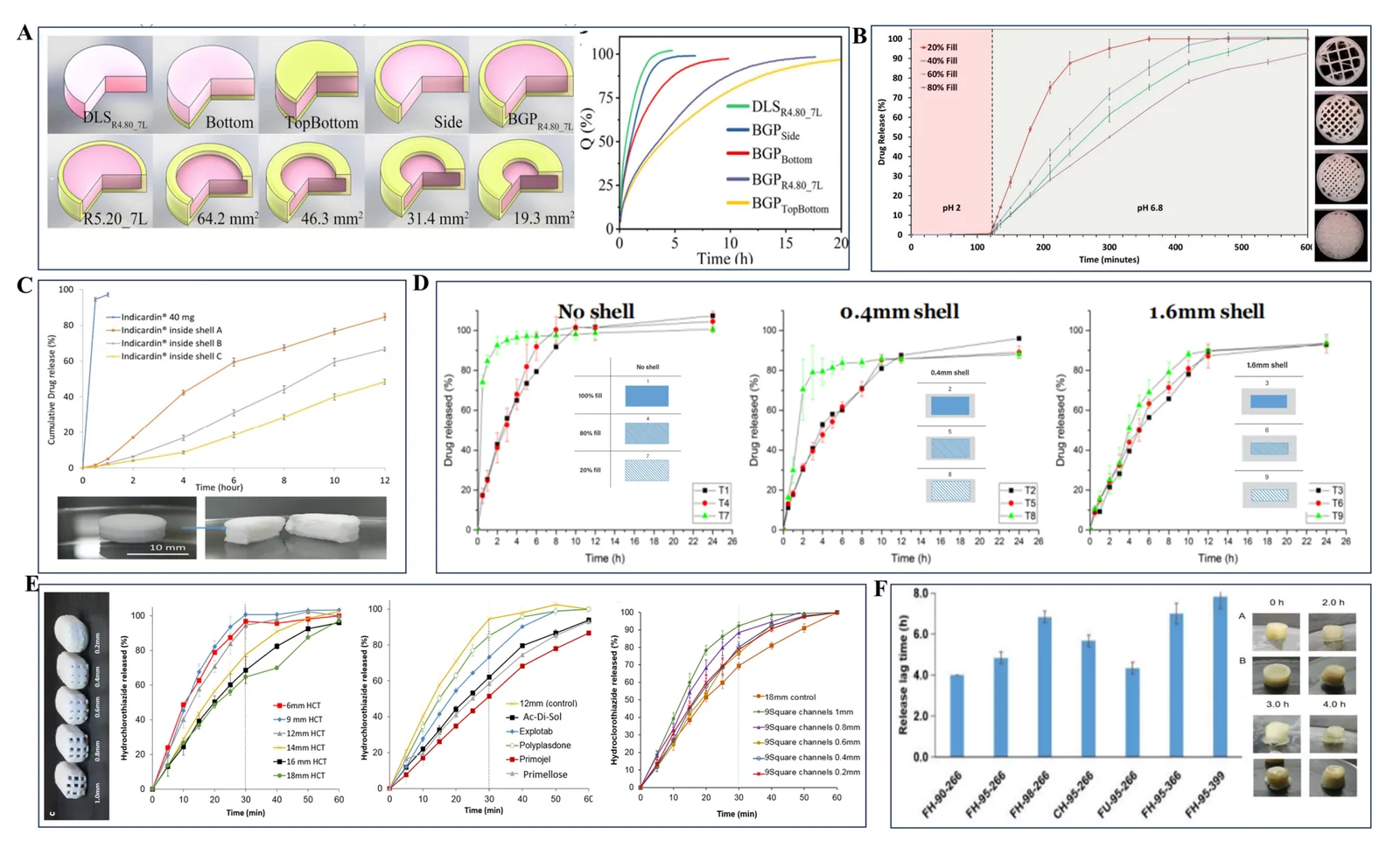
Representative 3D-printed sustained- and controlled-release formulations. (**A**) Multi-material DIW printlets with programmable diffusion pathways, effective release areas, and internal kinetic gates enabling sustained and multistage drug release [176]. (**B**) HME-FDM printlets showing packing-density-dependent modulation of drug release without substantial changes in formulation composition [9]. (**C**) Semi-solid extrusion-printed cellulose acetate shell structures exhibiting shell composition- and porosity-dependent release control [10]. (**D**) HPMC-based controlled-release printlets demonstrating the combined effects of matrix composition and printed geometry on sustained and near zero-order release [73]. (**E**) Channel-containing FDM printlets in which channel width, length, and position regulate medium penetration, diffusion pathways, and drug release rates [66]. (**F**) FDM-printed HPMC/PEG 400 core–shell printlets with adjustable lag times for delayed or pulsatile release [177]. These examples illustrate how structural variables, including diffusion path length, shell barriers, packing density, geometry, and channel architecture, can be programmed to achieve predictable sustained, delayed, pulsatile, or near zero-order release profiles. Adapted with permission from the original publications.

For sustained- and controlled-release printlets, structural design primarily aims to regulate fluid penetration, extend diffusion pathways, maintain structural integrity, and control release timing. Compared with conventional matrix or coated systems, 3D printing allows drug distribution, infill density, shell thickness, pore size, openings, channels, and core–shell or multilayer architectures to be directly programmed into dosage-form design [64,127]. Early studies showed that printed structures could function as tunable diffusion barriers. Chen et al. used multi-material DIW to construct printlets containing drug-loaded regions, barrier-guided domains, and path-modulating structures. This strategy enabled the programming of diffusion path length, effective release area, and internal kinetic gates to achieve predictable sustained and multistage release profiles [176]. Algahtani et al. used semi-solid extrusion to construct cellulose acetate shells and regulated drug release by modifying shell composition and porosity [10]. Thakkar et al. further demonstrated that HME-FDM printlets could modulate release through changes in packing density without substantially altering formulation composition [9]. Collectively, these studies established shell design and infill density as direct structural variables for controlling medium penetration and diffusion distances. Core–shell and internal-geometry designs further expanded structural control from regulating release rate to tailoring release timing and profile shape. Li et al. used FDM-printed HPMC/PEG 400 shells surrounding verapamil hydrochloride cores to adjust lag time within 4–8 h, demonstrating the potential of printed structures for delayed or pulsatile release [177]. Xu et al. designed PVA structures with Cylinder, Horn, and Reversed Horn geometries and obtained constant, increasing, or decreasing release profiles after loading drug-containing gel into these structures [122]. These examples show that internal geometry can regulate not only the overall release rate but also the temporal evolution of drug release. Gel-forming matrices and internal channels provide further control over hydration behavior and diffusion pathways. Zhang et al. prepared HPMC-based controlled-release printlets and showed that matrix composition and printed geometry jointly influenced release behavior, with certain designs approaching zero-order release kinetics [73]. Cheng et al. further highlighted the importance of polymer viscosity, gel-layer formation, and diffusion pathways in HPMC hydrogel printlets [178]. Sadia et al. showed that channel width, length, and position could modulate medium penetration and release rate [66]. Representative 3D-printed sustained- and controlled-release formulations utilizing different structural design strategies are illustrated in Figure 3.

### 4.3. Site-Specific Formulations for the Gastrointestinal Tract

**Figure 4:**
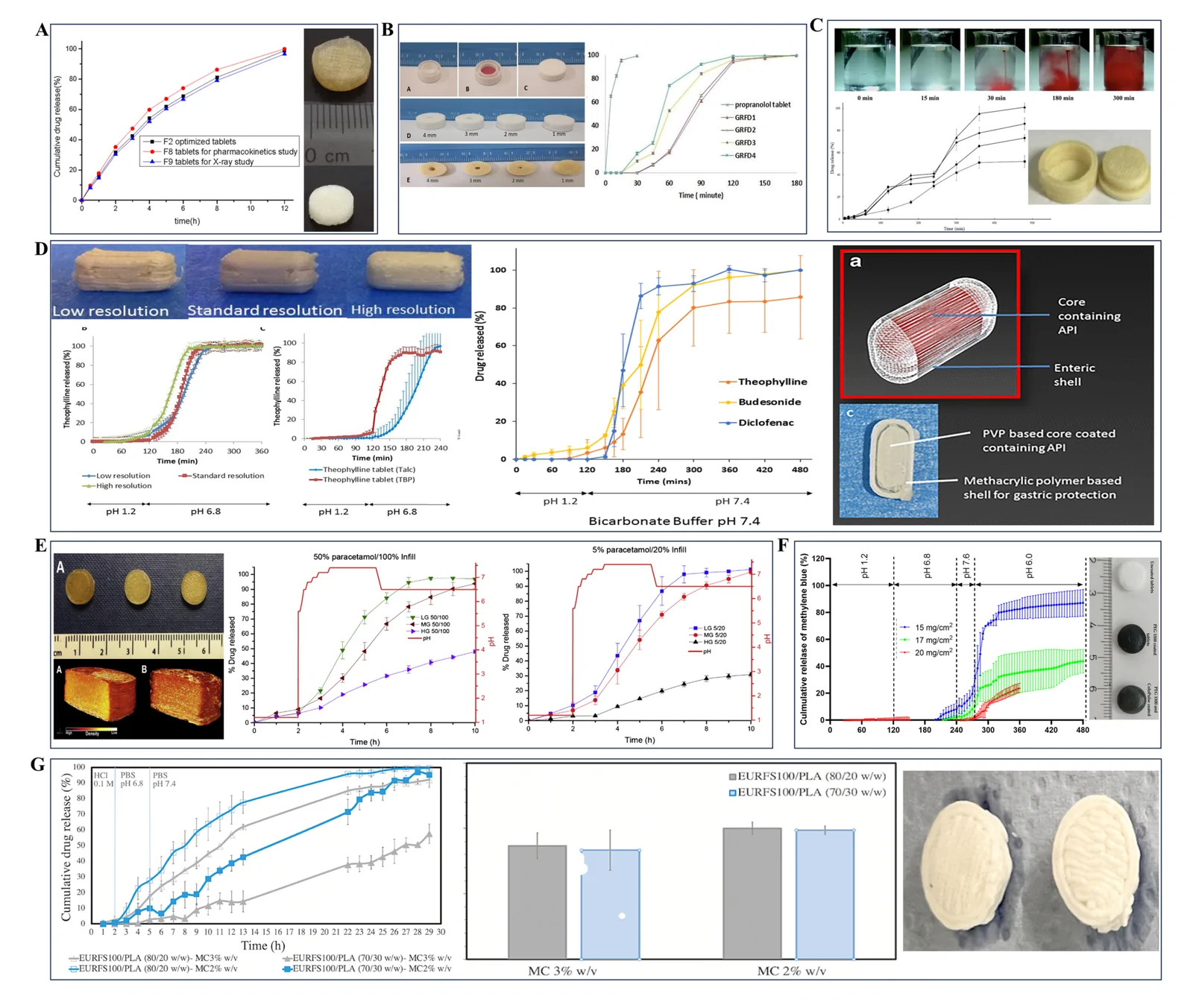
Representative 3D-printed gastrointestinal site-specific drug-delivery systems. (**A**) Hollow domperidone gastroretentive printlets with adjustable shell structures and packing density [180]. (**B**) Dual-chamber propranolol floating devices containing an air cavity and release opening for buoyancy and controlled drug release [181]. (**C**) Metronidazole gastroretentive printlets with tunable shell structures, orifices, and air chambers [182]. (**D**) Dual-nozzle PVP/Eudragit L100-55 core–shell printlets designed for gastric protection and delayed intestinal release [11]. (**E**) HPMCAS-based printlets exhibiting polymer-dependent delayed release [102]. (**F**) Alkaline phosphatase-loaded printlets developed for ileal-colonic delivery [184]. (**G**) Eudragit FS100/PLA shell–hydrogel core systems for distal intestinal or colon-oriented release [183]. Adapted with permission from the original publications.

Gastrointestinal site-specific printlets aim not only to prolong drug release but also to coordinate drug delivery with gastric residence, intestinal pH, transit time, and regional exposure. Three-dimensional printing enables this objective by integrating buoyancy structures, hollow cavities, pH-responsive barriers, time-control layers, openings, and compartmentalized architectures into a single dosage form [179]. Therefore, gastrointestinal delivery is determined not only by polymer responsiveness but also by how structural features regulate fluid penetration, floating behavior, and release onset. Gastroretentive systems provide a representative example of structure-enabled site-specific delivery. Chai et al. prepared domperidone gastroretentive printlets using HME-FDM and employed hollow structures, shell layers, and packing density adjustments to reduce density, achieving prolonged in vitro flotation and gastric retention in rabbits [180]. This study showed that low-density architecture can integrate buoyancy control with sustained release. Alqahtani et al. further incorporated immediate-release propranolol HCl tablets into an FDM-printed dual-chamber floating device, in which the air cavity provided buoyancy and the central opening regulated drug release [181]. Huanbutta et al. similarly used shell geometry, release orifices, and air chambers to modulate metronidazole flotation behavior and achieve near-zero-order release [182]. Collectively, these studies suggest that gastroretentive printlets can regulate both residence behavior and drug release through hollow structures, shell design, openings, and matrix composition. Representative 3D-printed gastrointestinal site-specific drug delivery systems, including gastroretentive and intestinal-targeted formulations, are illustrated in Figure 4.

Enteric and distal intestinal delivery systems rely primarily on pH-responsive materials combined with spatial barrier design. Okwuosa et al. used dual-nozzle FDM to fabricate shell-core gastric-resistant printlets consisting of a PVP-based drug-loaded core and an Eudragit L100-55 shell, demonstrating that printed enteric barriers with tunable shell thickness can protect the drug-containing core under acidic conditions [11]. Goyanes et al. further showed that HPMCAS grade and loading ratio influenced delayed release, indicating that both polymer pH responsiveness and internal structural design contribute to the regulation of release initiation and release rate [102]. For more distal intestinal or colon-oriented delivery, Asadi et al. constructed Eudragit FS100/PLA shell structures containing a methylcellulose hydrogel core [183], while Nguyen et al. developed alkaline phosphatase-loaded printlets for ileal-colonic delivery studies [184]. These examples extend the design concept from gastric protection toward pH-triggered, compartmentalized, and region-specific release. However, pH responsiveness or in vitro flotation performance alone cannot guarantee site-specific release in vivo, because gastrointestinal pH, transit time, food status, disease conditions, and interindividual variability may affect release onset and extent. Future studies should therefore combine dynamic dissolution models, structural integrity assessment, in vivo relevance assessment, and pharmacokinetic validation rather than relying only on simplified in vitro pH-switching or flotation tests.

### 4.4. Multicomponent Formulations Achieving Spatial Separation and Sequential Release

**Figure 5:**
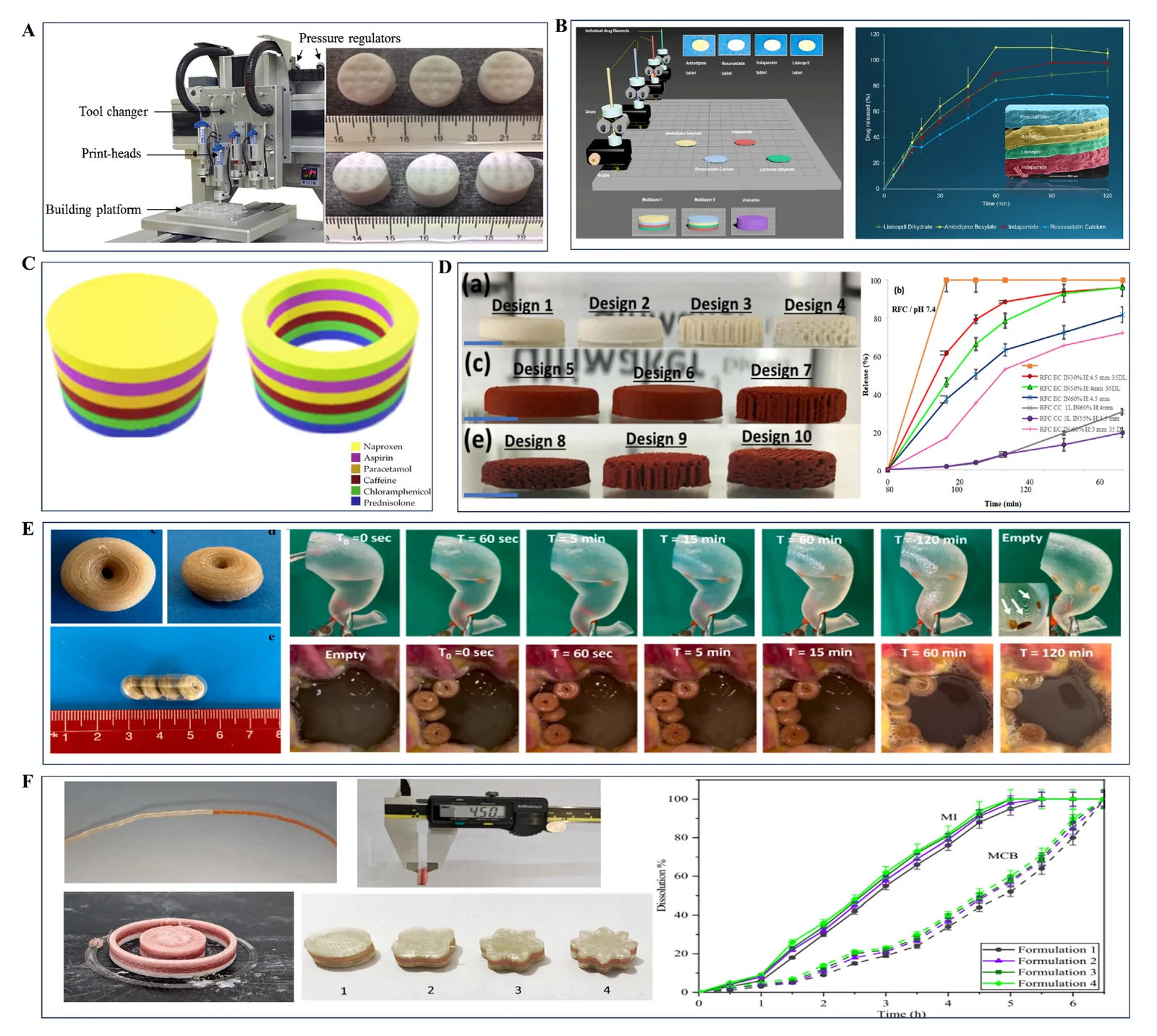
Representative 3D-printed multicomponent and sequential- release systems. (**A**) Five-drug cardiovascular polypill combining immediate and sustained release [3]. (**B**) PVA-based four-drug multilayer printlet exhibiting position-dependent release [114]. (**C**) Six-drug multilayer polypill showing differentiated release and interlayer drug migration [89]. (**D**) Dual-layer isoniazid-rifampin printlet enabling pH-dependent release separation [186]. (**E**) Floating antihypertensive polypill combining gastric retention and sustained release [187]. (**F**) Multilayer myo-inositol/methylcobalamin printlets exhibiting sequential release behavior [188]. Adapted with permission from the original publications.

Multicomponent and sequential-release systems represent important applications of pharmaceutical 3D printing. Conventional combination formulations mainly rely on co-mixing, layered compression, or capsule filling, which limits the control of drug compatibility, dose ratio, spatial distribution, and release sequence. In contrast, 3D printing enables different drugs to be organized into separate layers, compartments, shells, cores, or material domains, thereby achieving physical separation, differentiated release, and personalized dose combinations [185]. Therefore, the value of printed polypills lies not simply in incorporating multiple drugs into a single dosage form, but in assigning each drug to a defined structural region and release phase. Early studies demonstrated the feasibility of this concept. Khaled et al. developed a cardiovascular five-in-one polypill containing hydrochlorothiazide, ramipril, aspirin, pravastatin, and atenolol, achieving both immediate and sustained release through structural compartmentalization [3]. Pereira et al. further prepared a PVA-based four-drug multilayer printlet containing ramipril, indapamide, rosuvastatin, and amlodipine, showing that layer arrangement and drug positioning could influence multicomponent release kinetics [114]. Representative 3D-printed multicomponent formulations designed to achieve spatial drug separation and sequential release are illustrated in Figure 5.

Subsequent studies further highlighted that multicomponent printing must address not only drug organization but also drug migration and interfacial stability. Robles-Martinez et al. prepared a six-drug multilayer polypill and achieved differentiated release profiles; however, interlayer diffusion of some drugs was observed, indicating that spatial separation may not be fully maintained after printing or during storage [89]. This finding emphasizes that printed polypills require not only structural design but also careful consideration of material compatibility, interfacial control, and long-term stability evaluation.

More advanced designs have incorporated material partitioning and physiological triggers to minimize incompatibility and coordinate drug release. Ghanizadeh Tabriz et al. developed a dual-layer printlet containing isoniazid and rifampin, in which different matrices enabled the two anti-tuberculosis drugs to be released under different pH conditions, thereby reducing potential drug–drug interaction risks [186]. Zgouro et al. further combined a multidrug antihypertensive formulation with a hollow floating structure, integrating co-administration, gastric retention, and sustained release within a single printlet [187]. Reddy and Sharma fabricated multilayer polypills by thermally fusing different drug-loaded filaments. The resulting myo-inositol/methylcobalamin layered printlets exhibited position-dependent sequential release, demonstrating a simple strategy for achieving temporal control in multidrug formulations [188]. However, quality evaluation for these systems is more complex than that for single-drug printlets, involving content uniformity, cross-contamination, drug migration, material compatibility, release synchronization, stability, and clinical relevance. Future studies should therefore move beyond demonstrating the feasibility of printing multiple drugs within a single dosage form and provide evidence that such designs can improve adherence, therapeutic efficacy, safety, or clinical decision-making.

To further illustrate the relationship between digitally defined structural parameters and drug-release performance, representative quantitative structure–release findings from the studies discussed above are summarized in Table 4.

**Table 4:** Representative quantitative structure–release relationships in 3D-printed dosage forms.

Structural Variable	Printing Method/Formulation	Quantitative Structure–Release Finding	Structure–Release Implication	Study
Infill and shell configuration	FDM; PEO gastroretentive printlet	The optimized two-shell, 0% infill formulation remained floating for >10 h while providing extended drug release.	Hollow/low-infill architectures reduce apparent density and can integrate prolonged buoyancy with controlled release.	[75]
Core–shell barrier architecture	FDM; HPC/EC core–shell gastroretentive printlet	Structural design enabled a programmable lag time of approximately 30 min–6 h before drug release.	Shell barriers can regulate medium ingress and provide tunable delayed or pulsatile release.	[30]
Gap/fragmentation architecture	FDM; HPC gaplet	The gap-containing structure disintegrated within 4 min, with 86.7% drug released at 30 min.	Introducing structural discontinuities can accelerate fragmentation, medium penetration, and immediate drug release.	[108]
Core–shell osmotic architecture	FDM; PVA/CA/PVP core–shell printlet	Modification of the printed osmotic structure enabled release onset to be adjusted from approximately 0 to 360 min.	Structural barriers and controlled liquid-entry pathways can regulate release initiation independently of drug dose.	[110]
Number of shells	FDM; PETOx/PLA printlet	For DXM-loaded printlets, the 1-shell formulation released >80% of the drug within 60 min, whereas the 5-shell formulation released <70%. For HCTZ-loaded printlets, the corresponding release values were >60% and <40%, respectively.	Increasing the number of shell layers increases the effective diffusion barrier and decreases the release rate.	[116]
Dosage-form size	FDM; Eudragit EPO/PEO caplet	The smallest caplet released >80% of zolpidem within approximately 60 min, whereas the largest caplet required more than 200 min to reach >80% release.	Increasing dosage-form dimensions lowers the surface-area-to-volume ratio and lengthens diffusion pathways, thereby slowing release.	[120]
Hollow/floating architecture	FDM; PVA/EVA gastroretentive polypill	The floating mini-polypill released 75% of LD/BZ within approximately 750 min while maintaining prolonged drug release under gastric-retentive conditions.	Low-density gastroretentive architectures can combine buoyancy with prolonged drug release over 24 h.	[121]
Shell composition, geometry, and thickness	FDM; HPMC/PEG 400 core–shell system	Modification of the printed shell enabled the release lag time to be adjusted over approximately 4–8 h.	Shell geometry and diffusion-path length can be digitally programmed to control delayed or pulsatile release.	[177]
Layer number/surface-area-to-volume ratio	DIW/SSE; Kollicoat^®^ IR printlet	Increasing the number of printed layers progressively decreased the release rate; release behavior was correlated with the surface-area-to-volume ratio.	Layer number alters exposed surface area and diffusion distance, providing a direct structural handle for release-rate adjustment.	[143]
Core–shell diffusion barrier	DIW; PVP K90/Eudragit^®^ RS–RL system	The printed core–shell system sustained release of the highly water-soluble drug for >12 h.	A diffusion-controlling external shell can substantially extend release even for highly soluble, high-dose drugs.	[59]
Internal porosity/binder-generated structure	Binder jetting; MCC/lactose/mannitol/PVP K30 orally disintegrating tablets	All printed tablet specifications disintegrated within 10 s and achieved almost complete drug release within 5 min.	Highly porous binder-jetted architectures promote rapid liquid penetration, disintegration, and drug release.	[150]
Multilayer spatial organization	SLA; PEGDA multilayer system	At 20 h, paracetamol, caffeine, and aspirin reached approximately 100% release, chloramphenicol and naproxen exceeded 80%, whereas prednisolone reached approximately 45%.	Spatially separated multilayer architectures can accommodate drugs with distinct release profiles within a single printed system.	[89]

Note: CA, cellulose acetate; DIW, direct ink writing; EC, ethyl cellulose; FDM, fused deposition modeling; HPC, hydroxypropyl cellulose; PEO, polyethylene oxide; PETOx, poly(2-ethyl-2-oxazoline); PLA, polylactic acid; PVA, polyvinyl alcohol; SSE, semi-solid extrusion. Quantitative values represent the experimental conditions reported in the cited studies and should not be interpreted as universal relationships applicable to all material–process combinations.

### 4.5. Section Summary and Perspective

Across immediate-release, controlled-release, gastrointestinal site-specific, multicomponent, and localized delivery systems, the common value of 3D printing lies in transforming structural parameters into controllable variables for drug release [5,64]. By adjusting porosity, infill density, shell thickness, pore size, openings, material partitioning, and spatial drug distribution, printed dosage forms can regulate fluid penetration, matrix hydration or degradation, diffusion distance, exposed surface area, and release timing (Table 5) [10,63,127]. Therefore, structure-enabled formulation design should move beyond simply demonstrating that complex geometries can be fabricated. Future studies should establish interpretable and quantitative relationships among structural parameters, material properties, printing processes, and release performance [17,20,64]. This is particularly important for translating porous rapid-release systems, shell- or channel-controlled sustained-release systems, gastrointestinal site-specific formulations, and multicomponent printlets from empirical case studies into reproducible design strategies. Only when structural design becomes predictable, verifiable, and manufacturable can printed dosage forms advance toward scalable and clinically translatable drug delivery systems [13,101].

**Table 5:** Release objectives, structural variables, and technical adaptations for 3D-printed dosage forms.

Formulation Type	Primary Release Objectives	Key Structural Variables	Common Materials	Suitable Technologies	Key Advantages	Key Challenges
Immediate-release/ rapid-disintegrating oral formulations	Rapid wetting, disintegration, and drug release	High porosity, low infill density, thin layers, high surface-area-to-volume ratio	PVA, PVP, Kollidon VA64, sugar alcohols, cyclodextrins	BJ, SLS, DIW, FDM	Suitable for patients with dysphagia and individualized dosing	Mechanical strength, moisture absorption, and packaging protection
Sustained-release/controlled-release formulations	Prolonged release and reduced peak plasma concentration	Coating thickness, pore structure, infill density, core–shell structure	HPMC, HPC, EC, CA, PCL, PLA	FDM, DIW, SLS	Adjustable release profiles and suitability for long-term drug delivery	Challenges in structural consistency and release predictability
Gastroretentive formulations	Prolonged gastric retention and localized drug release	Hollow and low-density structures, porous, floating shells	HPMC, PVA, PLA, EVA	FDM, DIW	Adjustable buoyancy and release profiles	Significant variation in in vivo gastric emptying
Enteric/ colon-targeted formulations	Site-specific gastrointestinal release	pH-responsive coatings, multilayer barriers, compartmentalized structures	Eudragit L/S/FS, HPMCAS	FDM, DIW, SLS	Enables acid protection and intestinal release	Variations in in vivo pH and gastrointestinal transit time
Multicomponent formulations	Spatial separation and time-controlled release of multiple drugs	Multilayer, multi-compartment, core–shell, and compartmentalized drug loading	PVA, HPMC, Eudragit, PLA, etc.	FDM, DIW, SLA/DLP	Reduces medication burden and supports personalized combinations	Content uniformity, compatibility, and release profile matching

Note: BJ, binder jetting; CA, cellulose acetate; DIW, direct ink writing; DLP, digital light processing; EC, ethyl cellulose; EVA, ethylene-vinyl acetate copolymer; FDM, fused deposition modeling; HPC, hydroxypropyl cellulose; HPMC, hydroxypropyl methylcellulose; HPMCAS, hydroxypropyl methylcellulose acetate succinate; PCL, polycaprolactone; PLA, polylactic acid; PVA, polyvinyl alcohol; PVP, polyvinylpyrrolidone; SLA, stereolithography; SLS, selective laser sintering.

## 5. From Printability to Translatability: Remaining Barriers and Future Requirements

Clinical translation requires process-compatible excipients, efficient formulation development, validated release prediction, consistent product quality, and clinically relevant evidence [5,13]. The following sections examine these barriers and the manufacturing and regulatory requirements for addressing them.

### 5.1. Pharmaceutical Excipients Require Printing-Oriented Evaluation and Design

The limited availability of pharmaceutical excipients with validated printability, safety, and release-regulating functions remains a fundamental challenge in pharmaceutical 3D printing [15]. Most existing excipients were originally developed for conventional manufacturing processes, including compression, granulation, coating, capsule filling, and injection molding. Accordingly, their functional evaluation has primarily focused on properties such as flowability, compressibility, disintegration, film formation, and stability. In contrast, 3D printing is a layer-by-layer, path-dependent, and predominantly non-compressive manufacturing process that requires materials to undergo continuous formation under specific thermal, rheological, powder-processing, or photopolymerization conditions [15,26,42]. Consequently, although traditional excipients may demonstrate established pharmaceutical safety, they may not necessarily provide reliable printability within a specific additive-manufacturing process. Conversely, printable materials may not simultaneously satisfy requirements for pharmaceutical safety, drug release regulation, and industrial acceptability [15,101]. This mismatch manifests differently across printing technologies. FDM requires thermoplastic processability and sufficient filament robustness [16]. DIW requires precisely controlled rheological properties and sufficient shape retention after extrusion [33,82]. BJ and SLS require appropriate powder flowability, spreading performance, and consolidation behavior, while also facing challenges related to powder recovery, dust control, and management of residual unconsolidated powder. Photopolymerization-based systems additionally require pharmaceutical-grade photoreactive materials with acceptable residual profiles and sufficient drug photostability [41,189]. Therefore, the material challenge in pharmaceutical 3D printing is not simply the limited availability of printable polymers. More fundamentally, the field lacks a unified material-design framework that integrates pharmaceutical suitability, printability, drug-delivery functionality, and translational feasibility. Future development should proceed along two parallel directions. One direction is to systematically re-evaluate existing pharmaceutical excipients under printing-relevant conditions, including processability, structural retention, drug stability, and release behavior. The other is to develop printing-oriented excipient systems that can simultaneously satisfy requirements for safety, process compatibility, release regulation, and manufacturability.

### 5.2. Formulation and Process Development Require Data-Driven Design

Pharmaceutical 3D printing is often described as a digital manufacturing technology; however, in many studies, digitalization remains largely limited to model construction and digitally defined geometry fabrication, whereas material screening, process optimization, and release regulation still rely heavily on empirical expertise [17,20]. Development typically begins with the screening of printable materials, followed by repeated adjustment of drug loading, plasticizer ratio, solvent composition, ink rheology, filament mechanical properties, powder spreading conditions, or photopolymerization parameters. Without systematic design of experiments (DoE) or data-driven modeling, these adjustments often represent case-specific optimization rather than transferable design principles [15,26,82]. The fundamental reason is that the performance of printed dosage forms is jointly governed by materials, drugs, processing conditions, and structural design. During printing, factors such as thermal exposure, shear stress, interlayer bonding, pore formation, drying shrinkage, and curing degree can influence medium penetration, material hydration, drug diffusion, and release behavior [16,33,42]. In addition, variables including packing density, surface-to-volume ratio, shell thickness, pore size, polymer grade, and plasticizer systems can significantly alter release profiles. However, the relationships among these variables are often established only within specific material-process combinations, limiting their applicability to new active ingredients, polymer systems, or printing platforms [16,42]. This empirically driven development paradigm restricts the efficiency, reproducibility, and scalability of printed dosage forms. Future efforts should integrate experimental design, response surface methodology, machine learning, rheological analysis, thermal characterization, imaging techniques, and release data to establish predictive frameworks that link formulation composition and printing parameters with drug-delivery performance. In this context, intelligent pharmaceutical 3D printing should extend beyond automated geometry generation. It should enable pre-printing prediction of material processability, structural stability, and release behavior, while supporting iterative optimization through data-driven feedback.

### 5.3. Structure-Aware Release Prediction Remains a Core Scientific Challenge

Release prediction is essential for advancing pharmaceutical 3D printing from structural innovation toward transferable design principles. Traditional models, including zero-order, first-order, Higuchi, and Korsmeyer-Peppas models, are widely used to describe release profiles and identify underlying mechanisms. However, these models are generally more suitable for systems with relatively homogeneous compositions and simple geometries. In multilayered, core–shell, multicompartment, porous, or multi-material printlets, drug release is governed by the combined effects of material composition, spatial architecture, pore connectivity, medium-entry pathways, drug distribution, and dynamic structural evolution. Such complex interactions are difficult to capture using a single empirical model [10,64,66]. For example, reducing infill density typically increases porosity, shortens diffusion pathways, and accelerates drug release. However, in swelling or erosion-based systems, these pathways continuously change during hydration and gel-layer formation, meaning that release pathways are not static [63,127]. In non-erodible barrier systems, shell thickness, open pore area, and effective diffusion distance may dominate release behavior, whereas in hydrophilic gel-forming systems, hydration, swelling, and erosion interact with geometric features to determine release profiles [10,73]. Therefore, drug release from 3D-printed formulations is not controlled by a single material or structural parameter but represents a dynamic process governed by the interdependent effects of material properties, structural characteristics, and processing conditions. Future release prediction should move beyond traditional curve fitting toward structure-aware modeling approaches. Several computational tools provide complementary approaches across different spatial scales. Finite element analysis (FEA) can discretize complex dosage-form geometries to simulate diffusion, mass transfer, deformation, and mechanical responses, thereby enabling direct incorporation of structural variables such as shell thickness, pore architecture, and spatial material distribution [190]. Computational fluid dynamics (CFD) can further describe fluid penetration, convective transport, and drug mass transfer within and around structured dosage forms, and has been applied to model swelling, polymer dissolution, and drug diffusion in hydrophilic matrices [191]. Cellular automata provide a discrete modeling strategy in which local rules are used to simulate dynamic processes such as wetting, dissolution, diffusion, swelling, and erosion, making them particularly useful when the internal structure evolves during release [192]. At a smaller spatial scale, molecular dynamics (MD) simulations can characterize drug–polymer interactions, miscibility, molecular mobility, and cohesive behavior, providing molecular-level parameters that complement continuum-scale release models. These methods therefore address different aspects of the material–structure–release relationship rather than serving as interchangeable alternatives to empirical kinetic models [193,194].

A promising direction is to integrate three-dimensional structural parameters, material properties, process parameters, and in vitro release data within a unified framework that combines machine learning, mechanistic modeling, and computational pharmaceutics approaches to establish interpretable predictive systems. For complex printlets, reporting only the final release profile is insufficient. A minimum structural reporting framework should include infill patterns, porosity, shell thickness, opening area, spatial drug distribution, material partitioning, and structural evolution during dissolution [63]. Only when structural parameters can be quantitatively characterized and linked to release outcomes through transferable relationships can 3D-printed formulations progress from empirical design toward predictive design.

### 5.4. Quality Control and Scale-Up Require Process-Oriented Frameworks

The industrialization of 3D-printed pharmaceuticals requires more than simply increasing printing speed. It also depends on consistent product quality, process controllability, equipment qualification, and well-defined batch specifications. As structural complexity increases, product quality is determined not only by formulation composition but also by process variables, including print path, layer thickness, nozzle condition, platform temperature, ambient humidity, powder spreading behavior, light intensity, post-processing conditions, and equipment calibration [15,26,35]. Conventional quality attributes, including content uniformity, dissolution, mechanical strength, friability, residual solvents, impurities, and stability, remain essential for printed formulations. However, 3D printing introduces additional structure-related critical quality attributes, such as structural fidelity, pore connectivity, internal defects, interlayer bonding, path deviation, shell-thickness consistency, and spatial drug-distribution uniformity [64,127]. These attributes are closely associated with release behavior but are difficult to fully characterize through end-product testing alone. Future quality control strategies should therefore integrate in-process monitoring with structural characterization. Visual imaging, weight monitoring, thermal imaging, and near-infrared spectroscopy may enable real-time or near-real-time monitoring of printing processes, whereas micro-CT and image analysis can facilitate the evaluation of internal architecture, pore connectivity, shell thickness, and hidden defects. Production throughput represents an additional practical limitation for industrial translation [195]. Conventional high-speed rotary tablet presses can routinely produce more than 100,000 tablets per hour, with some industrial systems reaching several hundred thousand or even more than one million tablets per hour. In contrast, current pharmaceutical 3D-printing platforms generally operate at substantially lower throughputs because fabrication time depends on printing technology, dosage-form geometry, resolution, and the number of units produced simultaneously. Reported printing times for individual oral dosage forms can range from seconds to several minutes, and even parallel or multi-unit printing generally remains less competitive than rotary compression for high-volume manufacture. Therefore, the economic value of pharmaceutical 3D printing is unlikely to lie in replacing conventional tablet presses for mass production of standardized products. Its greater practical advantage may instead be found in small-batch, dose-flexible, personalized, or point-of-care manufacturing, where reduced tooling requirements, rapid digital design changes, and lower inventory of multiple fixed-dose strengths may partly offset lower production throughput. For scale-up, a process-oriented quality framework is required to define equipment qualification, process-parameter control, cleaning validation, batch-to-batch consistency, and product release criteria [196]. Accordingly, scale-up strategies should consider not only print speed but also parallelization, multi-nozzle or multi-unit fabrication, post-processing time, equipment utilization, labor requirements, and cost per acceptable dosage unit. Therefore, quality control for printed medicines should transition from end-product inspection toward a quality-by-design framework adapted to additive manufacturing processes and GMP requirements.

### 5.5. Clinical Translation and Regulatory Evidence Still Need to Be Strengthened

A printed product must demonstrate clear advantages in terms of patient acceptability, dosing accuracy, in vivo exposure control, therapeutic efficacy, safety, or manufacturing flexibility. The strongest clinical rationale for pharmaceutical 3D printing lies in applications where conventional fixed-dose products are insufficient, including pediatric and geriatric populations, patients with dysphagia, rare diseases, flexible dose-adjustment requirements, and chronic multidrug therapies. In these settings, dose flexibility, improved administration convenience, spatial drug separation, and coordinated release strategies may provide meaningful clinical benefits. However, for sustained-release, gastrointestinal site-specific, or implantable systems, differences observed in in vitro release profiles should be further correlated with improvements in pharmacokinetic exposure, localized drug delivery, therapeutic response, or safety. In vitro release performance alone should not be considered sufficient evidence of clinical value. Aprecia’s ZipDose^®^ platform, exemplified by the FDA-approved SPRITAM^®^ levetiracetam product, represents the most established example of commercial translation [197]. More recently, Triastek has advanced its Melt Extrusion Deposition (MED^®^) platform toward clinical development. MED^®^ was accepted into the FDA Emerging Technology Program in 2020, and multiple MED-based 3D-printed drug candidates have subsequently received FDA IND clearance, demonstrating progression from manufacturing-platform development toward clinical evaluation [198]. At the clinical and point-of-care level, Rodríguez-Pombo et al. evaluated personalized 3D-printed medicines in pediatric patients with rare metabolic disorders [32], illustrating the feasibility of incorporating dose-flexible additive manufacturing into clinically relevant personalized-treatment workflows.

Regulatory evaluation must determine whether printed medicines can be manufactured safely, consistently, and within a clearly defined design space. Required evidence should include material safety, process robustness, dose accuracy, structural fidelity, release consistency, stability, and in vivo performance. In the United States, the FDA Emerging Technology Program provides a mechanism for early interaction between sponsors and regulators regarding innovative manufacturing technologies and associated quality strategies [199]. The FDA also issued the Technical Considerations for Additive Manufactured Medical Devices guide in 2017, which established early principles for design, manufacturing, process validation, and testing of additively manufactured products, although its scope is limited to medical devices rather than pharmaceutical dosage forms [200]. More directly relevant to medicines, the FDA Center for Drug Evaluation and Research subsequently published the 2022 discussion paper Distributed Manufacturing and Point-of-Care Manufacturing of Drugs under the FRAME initiative, highlighting regulatory considerations related to manufacturing location, quality oversight, control strategies, and responsibilities across distributed or point-of-care sites [201]. In the United Kingdom, the Human Medicines (Amendment) (Modular Manufacture and Point of Care) Regulations 2025 established a dedicated regulatory framework for modular and point-of-care manufacture, including highly personalized or decentralized medicinal products [202]. In Europe, the EMA Quality Innovation Group issued Questions and Answers on the Implementation of 3DP Technology for Solid Oral Dosage Forms in 2026, addressing quality considerations including material attributes, process parameters, content uniformity, dissolution, porosity, stability, structural characteristics, and patient-centred dosage-form design [203]. Adjustable-dose or point-of-care manufacturing introduces additional regulatory considerations, including digital model and version control, acceptable ranges for dose and structural modification, batch definition, equipment and site qualification, operator responsibility, traceability, and documentation requirements (Table 6).

**Table 6:** Challenges and future directions for clinical translation of pharmaceutical 3D printing.

Challenges	Main Manifestations	Root Causes	Future Directions
Insufficient pharmaceutical excipients	Limited printable materials and inconsistency between pharmaceutical suitability and printability	Traditional excipients were not developed for layer-by-layer manufacturing	Develop printing-specific pharmaceutical excipients and establish excipient compatibility evaluation systems
Formulation development relies on trial and error	Low efficiency in material screening, parameter optimization, and release control	The relationships among materials, processes, structures, and performance remain unclear	Apply DoE, response surface methodology, machine learning, and data-driven formulation design
Difficulty in predicting release	Complex structural release mechanisms are difficult to interpret using traditional models	Complex release pathways caused by multilayer structures, channels, shells, and material partitioning	Develop structure-aware release models and standardized structural descriptors
Inadequate quality control	Batch-to-batch consistency, internal structures, and interlayer quality are difficult to evaluate	Traditional inspection methods struggle to capture structure-related quality attributes	Implement online monitoring, process analytical technologies, micro-CT, and image analysis
Difficulties in scaling up manufacturing	Insufficient printing speed, equipment validation, and process stability	Single-unit manufacturing differs from conventional batch pharmaceutical production	Develop multi-nozzle systems, continuous manufacturing strategies, GMP-compliant processes, and equipment standardization
Limited clinical evidence	Structural advantages have not been fully translated into therapeutic benefits	Most studies remain at the in vitro release and proof-of-concept stages	Strengthen in vitro-in vivo correlation, pharmacokinetic studies, and evaluations of patient benefits

Note: DoE, design of experiments; GMP, good manufacturing practice; micro-CT, micro-computed tomography.

## 6. Conclusions

Pharmaceutical 3D printing enables polymer functions and spatial architecture to be jointly used in dosage-form design. Current evidence supports tunable immediate, sustained, delayed, and multidrug release within defined material and process windows, while gastrointestinal and localized delivery require additional biological validation. Priorities include printing-relevant excipient evaluation, quantitative characterization of the structures actually produced, independently validated release models, storage stability assessment, and feasible manufacturing workflows. Process monitoring and quality-by-design strategies must be accompanied by product-specific clinical and regulatory evidence. These advances can support reliable personalized and controlled-release medicines.

## Data Availability

No new data were created or analyzed in this study. Data sharing is not applicable to this article.

## Abbreviations

The following abbreviations are used in this manuscript:

ABS	Acrylonitrile butadiene styrene
AETMA	[2-(Acryloyloxy)ethyl]trimethylammonium chloride
ATPU	Aliphatic thermoplastic polyurethane
CA	Cellulose acetate
CAP	Cellulose acetate phthalate
CMCS	Carboxymethyl chitosan
EC	Ethyl cellulose
EVA	Ethylene-vinyl acetate copolymer
GelMA	Gelatin methacryloyl
HEC	Hydroxyethyl cellulose
HPC	Hydroxypropyl cellulose
HPMC	Hydroxypropyl methylcellulose
HPMCAS	Hydroxypropyl methylcellulose acetate succinate
HPβCD	Hydroxypropyl-β-cyclodextrin
MCC	Microcrystalline cellulose
PCL	Polycaprolactone
PEG	Polyethylene glycol
PEGDA	Poly(ethylene glycol) diacrylate
PEO	Polyethylene oxide
PETOx	Poly(2-ethyl-2-oxazoline)
PLA	Polylactic acid
PLGA	Poly(lactic-co-glycolic acid)
PTMC	Poly(trimethylene carbonate)
PVA/PVOH	Polyvinyl alcohol
PVP	Polyvinylpyrrolidone
SA	Sodium alginate
TPE-E	Thermoplastic polyester elastomer
TPU	Thermoplastic polyurethane
4-ASA	4-Aminosalicylic acid
5-ASA	5-Aminosalicylic acid
APF-DDM	Arburg plastic freeforming-droplet deposition modeling
bio-SNEDDS	Bioactive self-nanoemulsifying drug delivery system
BJ	Binder jetting
C6NP	Coumarin-6 nanoparticles
CLIP	Continuous liquid interface production
DEAM	Direct extrusion additive manufacturing
DIW	Direct ink writing
DLP	Digital light processing
DoE	Design of experiments
FDM	Fused deposition modeling
FFF	Fused filament fabrication
GMP	Good manufacturing practice
HME	Hot-melt extrusion
IgG	Immunoglobulin G
IVF	Injection volume filling
micro-CT	Micro-computed tomography
PAM	Pressure-assisted microsyringe
PK	Pharmacokinetics
SA/V	Surface area-to-volume ratio
SLA	Stereolithography
SLS	Selective laser sintering
SMEDDS	Self-microemulsifying drug delivery system
SSE	Semi-solid extrusion
TPP	Two-photon polymerization
